# Integration of individualized and population-level molecular epidemiology data to model COVID-19 outcomes

**DOI:** 10.1016/j.xcrm.2023.101361

**Published:** 2024-01-16

**Authors:** Ted Ling-Hu, Lacy M. Simons, Taylor J. Dean, Estefany Rios-Guzman, Matthew T. Caputo, Arghavan Alisoltani, Chao Qi, Michael Malczynski, Timothy Blanke, Lawrence J. Jennings, Michael G. Ison, Chad J. Achenbach, Paige M. Larkin, Karen L. Kaul, Ramon Lorenzo-Redondo, Egon A. Ozer, Judd F. Hultquist

**Affiliations:** 1 Department of Medicine, Division of Infectious Diseases, Northwestern University Feinberg School of Medicine, Chicago, IL 60611, USA; 2 Center for Pathogen Genomics and Microbial Evolution, Northwestern University Havey Institute for Global Health, Chicago, IL 60611, USA; 3 Clinical Microbiology Laboratory, Department of Pathology, Northwestern Memorial Hospital, Chicago, IL 60611, USA; 4 Diagnostic Molecular Biology Laboratory, Northwestern Memorial Hospital, Chicago, IL 60611, USA; 5 Havey Institute for Global Health, Feinberg School of Medicine, Northwestern University, Chicago, IL 60611, USA; 6 Department of Molecular Microbiology, Northshore University HealthSystem, Evanston, IL 60201, USA; 7 Department of Pathology, Northshore University HealthSystem, Evanston, IL 60201, USA

**Keywords:** SARS-CoV-2, molecular epidemiology, phylogenetics, viral evolution, genomic surveillance, variants of concern, COVID-19, severity modeling, confounders

## Abstract

Severe acute respiratory syndrome coronavirus 2 (SARS-CoV-2) variants with enhanced transmissibility and immune escape have emerged periodically throughout the coronavirus disease 2019 (COVID-19) pandemic, but the impact of these variants on disease severity has remained unclear. In this single-center, retrospective cohort study, we examined the association between SARS-CoV-2 clade and patient outcome over a two-year period in Chicago, Illinois. Between March 2020 and March 2022, 14,252 residual diagnostic specimens were collected from SARS-CoV-2-positive inpatients and outpatients alongside linked clinical and demographic metadata, of which 2,114 were processed for viral whole-genome sequencing. When controlling for patient demographics and vaccination status, several viral clades were associated with risk for hospitalization, but this association was negated by the inclusion of population-level confounders, including case count, sampling bias, and shifting standards of care. These data highlight the importance of integrating non-virological factors into disease severity and outcome models for the accurate assessment of patient risk.

## Highlights

• COVID-19 disease severity is associated with demographic and clinical risk factors
• Population-level variables can confound severity and outcome models
• Shifting standards of care, testing practices, and sampling skew outcome metrics

## Graphical abstract

**Figure:**
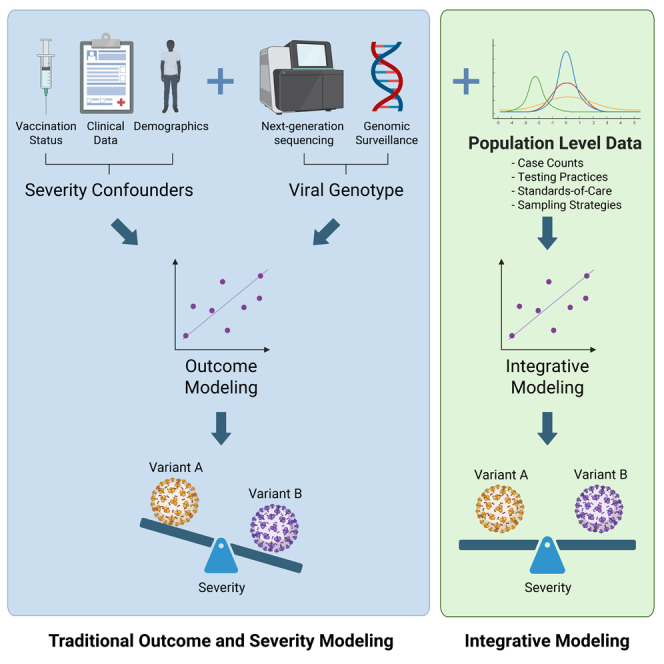

## Teaser

Ling-Hu et al. characterize the molecular epidemiology of the COVID-19 pandemic in Chicago over a 2-year period, integrating demographic, clinical, viral genetic, and epidemiological data. They find that population-level features, including shifting standards of care, can confound our understanding of disease severity and should be incorporated in future surveillance studies.

## Introduction

Since the beginning of the coronavirus disease 2019 (COVID-19) pandemic, the causative agent, severe acute respiratory syndrome coronavirus 2 (SARS-CoV-2), has continued to mutate and evolve over time, resulting in the emergence of different viral variants.^1^^,^^2^ Occasionally, these variations have conferred a fitness advantage to the virus, facilitating its spread.^3^^,^^4^^,^^5^ Variants that show evidence of rapid spread and increased transmissibility, enhanced immune evasion, or therapeutic resistance, are considered variants of concern (VOCs) by the World Health Organization (WHO).^6^ Genomic surveillance has been vital to tracking these variants, which has proved to be essential for informing clinical care practices, implementing public health measures, and communicating risks to the public.^7^

As the pandemic has proceeded, the medical and scientific communities have garnered a better understanding of the risk factors associated with severe COVID-19.^8^^,^^9^^,^^10^^,^^11^ Male gender, older age, and a number of pre-existing conditions/comorbidities such as high body mass index (BMI),^12^ cardiovascular disease,^13^ asthma,^14^ immunodeficiency,^15^^,^^16^ and diabetes^17^^,^^18^ have all been associated with more severe disease and worse patient outcomes. Social determinants of health such as access to health care, risk for exposure, and socioeconomic status have likewise been found to contribute to an individual’s disease risk.^17^^,^^19^^,^^20^ Nevertheless, increased natural immunity in the population alongside improvements in clinical care procedures and treatment options—including monoclonal antibody therapeutics, steroids, and antiviral drugs—have all dramatically improved patient outcomes over time.^21^^,^^22^^,^^23^^,^^24^ Most importantly, the rapid development and deployment of effective vaccines has greatly decreased morbidity and mortality due to COVID-19.^25^^,^^26^^,^^27^^,^^28^

Despite this improved understanding, it is unclear if and how different viral variants may affect disease severity and patient outcomes.^29^ One of the first mutations to have increased viral fitness was a D614G substitution in the viral Spike protein that increased viral infectivity and transmissibility.^30^^,^^31^^,^^32^ Although it increased viral loads in patient upper airways, it was not found to be associated with any change in disease severity or patient outcome.^33^ A number of reports have since ascribed worse outcomes or more severe disease to a number of subsequent VOCs, including Alpha,^34^ Beta,^35^ Delta,^36^ and Gamma,^37^ but these findings have not been widely confirmed.^38^ On the contrary, the Omicron VOC, which carries a highly divergent Spike protein, has been associated with altered disease presentation, lower disease severity, and decreased mortality across multiple studies, even when controlling for demographic and clinical cofounders, such as vaccination.^39^^,^^40^

Unfortunately, the collation of accurate patient outcome and severity data along with linked viral genomic data over a large enough population to properly power and control such studies is challenging and time consuming.^41^ Furthermore, understanding which confounders need to be modeled and assessed is critical to avoid spurious associations, especially when considering single-center studies. Depending on the availability and completeness of the associated metadata, most disease severity and outcome models incorporate individualized demographic and clinical features, including gender, age, vaccination status, and comorbidities.^42^^,^^43^ Although important, these features often fail to capture population-level changes that occur over the course of study, including changes in testing habits, clinical care practices, and surveillance protocols that may bias outcome models. This is particularly important when attempting to compare different viral variants that arose at different times.^44^ We hypothesize that inclusion of population-level epidemiological factors in a rigorous statistical model may help uncover non-virological factors that pertain to our understanding of severity.

To test this hypothesis, we performed a single-center, retrospective cohort study to examine the association between SARS-CoV-2 clade and patient outcome over a two-year period in Chicago, Illinois, USA. Chicago recorded its first case of COVID-19 on January 24, 2020.^45^ Within two years, the greater Chicago metropolitan area (Cook County) reported more than 1 million cases, 65,000 hospitalizations, and 14,000 deaths related to COVID-19. Early in the pandemic, we established a biobank of residual diagnostic specimens from adult patients who tested positive for SARS-CoV-2 at Northwestern Memorial Hospital (NMH) by PCR-based clinical testing for routine genomic surveillance. Data from electronic medical records for each specimen was collected retrospectively, including demographic data, pre-existing conditions, comorbidities, hospitalization records, administered therapeutics, vaccination history, and clinical lab values. In addition, population-level changes in epidemiological data were collated from the Chicago and Illinois departments of public health and NMH, including case counts, and tests administered. Here, we leveraged this dataset to assess the association between SARS-CoV-2 clade and clinical outcome in Chicago while controlling for non-virological features including individualized demographic and clinical data as well as population-level epidemiological factors.

## Results

### Timeline of the COVID-19 pandemic in Chicago

**Figure 1:**
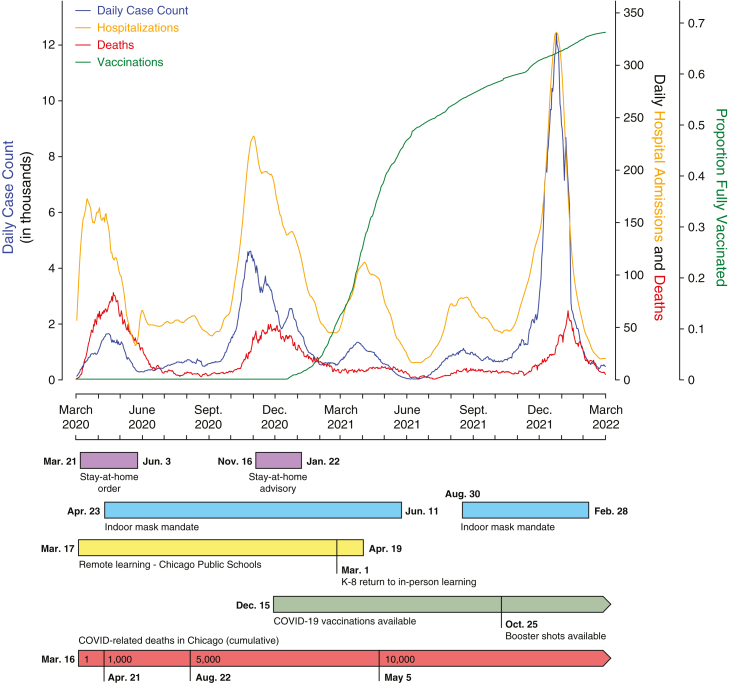
Summary of the COVID-19 pandemic in Cook County, Illinois Epidemiology of COVID-19 as represented by 7-day rolling average of cases (blue), hospitalizations (orange), and deaths (red) between March 17, 2020, and March 17, 2022. The cumulative fully vaccinated (1 dose of Johnson & Johnson or two doses of Pfizer or Moderna) proportion of the population is shown in green. A timeline of key public health mitigation measures is represented in color bars below. These same data for Illinois and the United States are shown in Figure S1.

To better understand the epidemiological timeline of the COVID-19 pandemic in Chicago, we extracted daily case count, hospitalization, death, and vaccination data for Cook County from the Illinois Department of Public Health (IDPH) from March 17, 2020, to March 17, 2022, and calculated a seven-day moving average for each variable (Figure 1).^46^ Hospitalization data before June 13, 2020, were not recorded by IDPH, thus hospitalization data from March 17, 2020, to June 12, 2020 were extracted from Chicago Department of Public Health (CDPH).^47^ These data revealed five distinct “waves” of elevated case counts and hospitalizations in the city over that two-year period. Select public health measures implemented in Cook County over that period are highlighted below the timeline (Figure 1).

Following the first case of SARS-CoV-2 in Illinois (reported on January 24, 2020), Chicago implemented several non-pharmaceutical interventions, including stay-at-home orders, indoor mask mandates, and remote learning, in an attempt to slow the spread of the virus. Subsequently, vaccinations were made widely available to the public in early 2021, with nearly 50% of Chicago’s population being fully vaccinated (defined as one dose of the US Food and Drug Administration [FDA]-approved adenovirus vaccine from Johnson & Johnson [JNJ-78436735] or two doses of an FDA-approved mRNA vaccine from Moderna [mRNA-1273] or Pfizer [BNT162b2]) by July 2021. Despite the authorization of booster shots for the COVID-19 vaccine in October 2021, cases increased rapidly in the winter of 2021, peaking at more than 12,000 new cases each day coincident with the emergence and spread of the Omicron VOC. Over this two-year period, more than 65,000 COVID-19-related hospitalizations and 14,000 COVID-19-related deaths were reported in Cook County.

City-level data closely reflected statewide (Figure S1A) and nationwide (Figure S1B) trends in case counts, hospitalizations, and deaths, though the greater United States saw an additional wave of cases in July 2020 that was not apparent in Chicago or Illinois. Case counts and hospitalization data were closely linked, though there were more hospitalizations and deaths relative to cases in earlier waves compared with later ones (Figures 1, S1A, and S1B). Although differences in public health measures, clinical care tools, population behavior, and vaccination played critical roles in decreasing the mortality and morbidity of COVID-19 in successive waves, it is unclear if the underlying viral variants responsible for each wave likewise differed in severity. Given that epidemiological trends in Chicago were broadly reflective of those across Illinois and the United States, we leveraged a retrospective biobank of SARS-CoV-2 clinical specimens collected in a single health care system in Chicago to test the association between viral clade and COVID-19 patient outcome while controlling for demographic, clinical, and epidemiological confounders.

### Viral whole-genome sequencing identifies unique variant distributions in each wave

In March 2020, we established a biobank of residual diagnostic specimens (primarily nasopharyngeal and oropharyngeal swabs) from adult patients who tested positive for SARS-CoV-2 by PCR-based clinical testing at NMH or at one of its affiliated drive-through testing centers, outpatient clinics, or immediate care clinics around the Chicago, Illinois, and suburban area. These specimens were retrospectively linked to demographic (gender, age, race, ethnicity, ZIP code, etc.) and clinical (BMI, hospitalization records, co-morbid conditions, vaccination history, clinical lab values, etc.) datasets for analysis. Between March 17, 2020, and March 17, 2022, we collected 14,252 SARS-CoV-2-positive specimens from unique adult patients (12,693 outpatients, 1,559 inpatients) living in Cook County (Table S1). The patient cohort skewed slightly female (57.6%) with a median age of 42 years (interquartile range [IQR], 26 years). Cohort race and ethnicity were broadly representative of the population of Chicago,^48^ though it was majority non-Hispanic (76.8%) and white (57.0%) (Table S1). The demographics of each wave were slightly different, with wave 1 skewing male (55.1%) and older (median age 56 years; IQR, 30 years), with fewer non-Hispanic (66.4%) and white (33.2%) patients.

Each month, a random subset of collected specimens (∼15%–20%) were selected for sequencing using a stratified randomization strategy, specifically stratifying for inpatient/outpatient status, date, and cycle threshold (Ct) value. Specimens with sufficient viral load (Ct value < 32 as determined by qRT-PCR) were subjected to SARS-CoV-2 whole-genome sequencing using a multiplex PCR amplicon strategy per the ARTIC protocol.^49^ In total, we obtained 2,114 whole-genome sequences with >90% coverage (Global Initiative on Sharing All Influenza Data [GISAID] accession numbers are available in Table S2). A post hoc analysis comparing the demographics of our sequenced cohort (n = 2,114) with that of our overall cohort (n = 14,252) showed that the demographics were roughly proportional by age, race, and gender, suggesting that a representative subset was sequenced (Table S1). Nextclade and Pangolin tools were used to assign clade and lineage designations, respectively.^50^^,^^51^ We use Nextclade designations throughout, but the Pangolin lineage equivalents are provided in Table S3. Given that variants ascribed a Greek alphabet moniker by the WHO have changed in status over time, we refer to them here by the most urgent status they were assigned (i.e., if a variant was designated a VOC at any point, it is referred to as such here).

**Figure 2:**
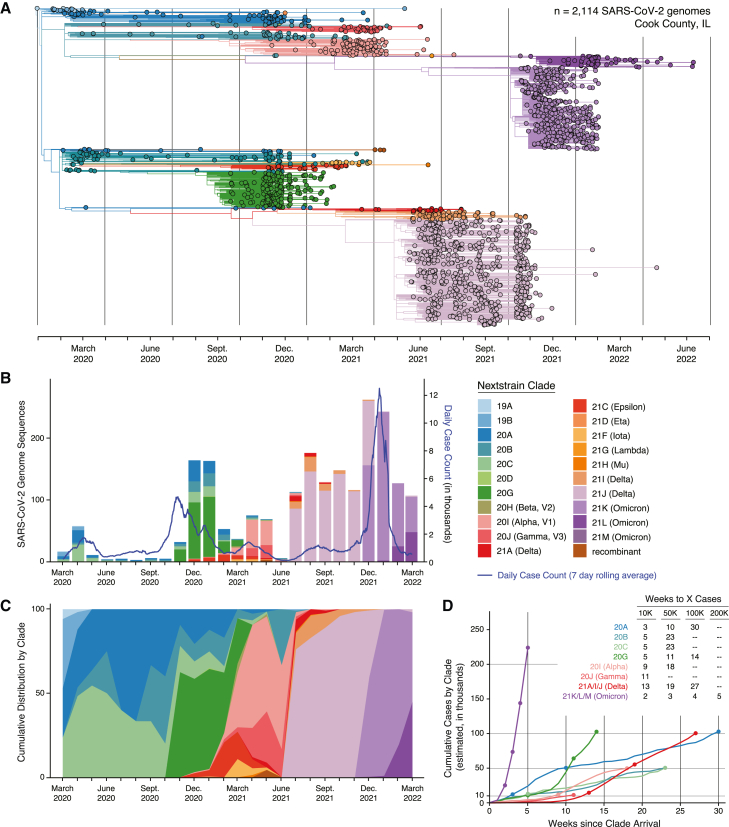
Genomic surveillance of SARS-CoV-2 in Cook County, Illinois (A) Estimated maximum likelihood phylogeny of SARS-CoV-2 genome sequences (n = 2,114). (B) Absolute count of sequences per month and associated clades reported in this study overlaid with the daily cases in Cook County. (C) Cumulative distribution of clade frequency over time in this study. (D) Estimated weeks to achieve cumulative case numbers per clade. Estimated cases were calculated by multiplying the frequency of clades by number of cases per week in each geographical region. See also Figure S2.

We used a maximum likelihood (ML) phylogenetic analysis to examine the continued evolution of SARS-CoV-2 in Chicago over time (Figure 2A). This analysis indicated that although a few early isolates belonged to the original 19A and 19B clades, these were quickly displaced by clades 20A, 20B, 20C, and 20G, harboring the transmission-enhancing Spike D614G mutation. These clades remained predominant until March 2021, with the emergence of primarily three different VOCs in Chicago: Alpha (20I), Gamma (20J), and Epsilon (21C). These were largely supplanted by the Delta VOC (21I/21J/21A) in July 2021 and subsequently by the Omicron VOC (21M/21K/21L) in December 2021. The number of sequenced isolates was largely proportional to case counts except for a period of relative oversampling between July and November 2021 (Figure 2B). These sequences are broadly representative of SARS-CoV-2 genetic diversity observed across the United States and the world, as shown in Figure S2A, though lacking in representation of some regionally concentrated VOCs that emerged in early 2021 prior to the global spread of the Delta VOC.

Only 5 clades or VOCs ever reached a majority (≥50%) of cases in Chicago: 20A in the summer of 2020, 20G in the winter of 2020, Alpha in the spring of 2021, Delta in the fall of 2021, and Omicron in winter of 2021–2022 (Figure 2C). Of these, only Delta and Omicron ever came near to full dominance, achieving 100% representation in our sampling during certain months. These data are very similar to the variant distributions calculated using all available sequence data from Chicago (Figure S2B). It likewise closely parallels the variant distributions observed in the relatively nearby city of Madison, Wisconsin. Comparing these data with data from other cities across the United States, variant distributions were largely similar with the exception of early 2021 when different VOCs dominated in different regions of the country (Figure S2B). Although Chicago saw a mix of Alpha, Gamma, and Epsilon VOCs, Houston (Texas) had primarily Alpha, Los Angeles (California) and Seattle (Washington) both had primarily Epsilon, and New York City (New York) had primarily Iota.

To further examine the expansion of each clade, we downloaded all publicly available sequences from GISAID collected in Cook County between March 17, 2020, and March 17, 2022 and calculated the weekly frequency of each clade.^52^ We then multiplied this frequency by the number of weekly cases, as reported by IDPH, to estimate the total number of cases caused by each clade following its introduction (i.e., after the first 10 cases were reported; Figure 2D). For practicality and ease of calculation, we pooled the separate Delta (21A, 21I, 21J) and Omicron (21K, 21L, 21M) clades together. Clade 21L (lineage BA.2) was just emerging in Chicago in March 2022 and lacked sufficient representation to be considered independently in this dataset. Any clade comprising less than 2% of total sequences (Mu [21H], Lambda [21G], Iota [21F], Eta [21D], Epsilon [21C], Gamma [20J], Beta [20H], 20D, 19B, 19A, or a recombinant clade) was pooled into a single “other” category. By this analysis, only 20A, 20G, Delta, and Omicron were estimated to have caused at least 100,000 cumulative cases in Cook County. However, while clade 20A took 30 weeks to cause this number of infections, Omicron surpassed 100,000 cases in only 4 weeks, doubling that count less than a week later. These 4 clades followed similar dynamics and were also estimated to be responsible for the highest cumulative case counts in the state of Illinois and across the entire United States (Figure S2C).

### Viral clade is associated with hospitalization when controlling for key demographic and clinical variables

**Figure 3:**
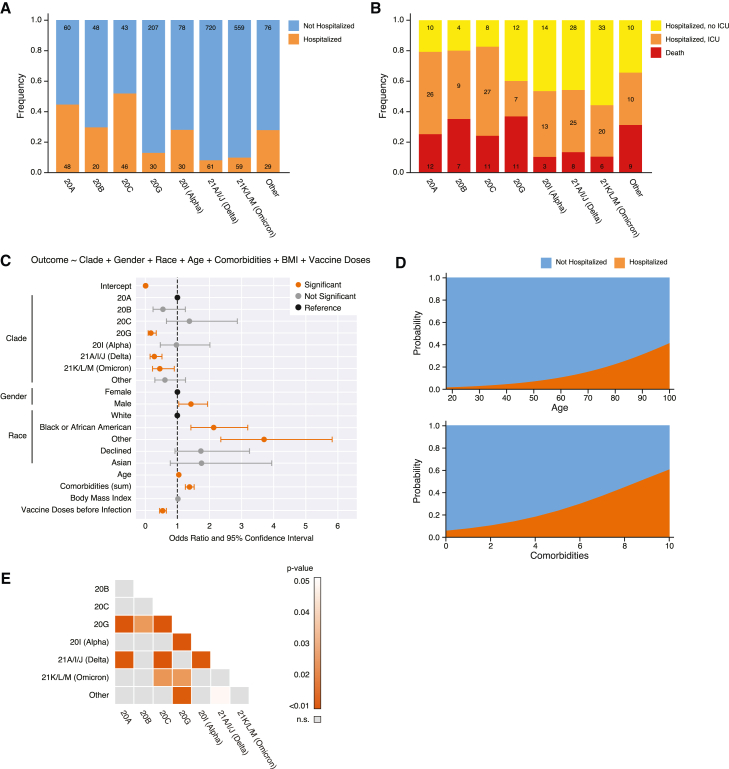
SARS-CoV-2 clade is associated with risk for hospitalization when controlling for demographics (A) Frequency of hospitalized (orange) versus non-hospitalized (blue) patients by clade (n = 2,114 patients). Absolute counts of patients are provided in each bar. (B) Frequency of hospitalized patients who were not admitted to the ICU (yellow), who were admitted to the ICU (orange), or who died (red) by clade (n = 323 patients). Absolute counts of patients are provided in each bar. (C) Odds ratio plot with 95% confidence intervals (CIs) as calculated by a multivariable logistic regression modeling risk for hospitalization (n = 1,599). Significant features (p < 0.05) are highlighted in orange. Model parameters are provided in Figure S3. (D) Effect plots showing the probability distribution of hospitalization when adjusting age and comorbidities for the model in (C). (E) Heatmap of adjusted p values following Benjamini-Hochberg correction to control for false discovery rate for pairwise comparisons of estimated marginal means between clades from the model in (C). See also Figure S3.

Based on the clinical record data corresponding to each sequenced specimen, we compared the frequency of inpatients (defined as individuals who were admitted to a hospital in the Northwestern Medicine health care network within 7 days of a documented positive COVID-19 molecular test result) and outpatients infected with each clade, again collapsing any clade that did not account for more than 2% of our specimens into a single “other” category (Figure 3A). Although higher hospital admission rates were observed among patients infected with clades 20A, 20B, 20C, and Alpha, hospital admission was less frequent for patients infected with clades 20G, Delta, and Omicron. We similarly compared the frequency of outcomes among inpatients divided into three mutually exclusive categories: hospital admission without intensive care unit (ICU) admission, ICU admission, and COVID-19-related death. Similar to hospitalization rates, the most severe outcomes were more frequent among patients infected with clades 20A, 20B, or 20C and less common among inpatients infected with clade 20G, Alpha, Delta, or Omicron (Figure 3B).

Several demographic and clinical variables have been previously associated with an increased risk for hospitalization, including gender, age, race, comorbidities, BMI, and vaccination status.^53^^,^^54^^,^^55^^,^^56^^,^^57^^,^^58^ To account for potential confounders in this analysis, we first used multivariable logistic regression to model clinical outcome (defined here as hospitalization versus no hospitalization) while controlling for gender, race, age, number of comorbidities, BMI, and the number of vaccination doses prior to infection (n = 11,007 patients with all available data; Figures S3A and S3B). Patients with any missing values were dropped from analysis for this and all subsequent models. Only vaccine doses administered at least 14 days before specimen collection date were considered “prior to infection,” with each shot representing one dose regardless of formulation (i.e., adenovirus or mRNA). To minimize error due to low sample numbers (<1% of the total dataset), we pooled the race categories of “Native Hawaiian or other Pacific Islander” and “American Indian or Alaska Native” into “other” and similarly collapsed “unknown” and “unable to answer” into “declined” (note that these categories reflect self-selected options and so were not considered as “missing” data fields). This model indicated that older age, higher BMI, more comorbidities, Black or African American race, and fewer vaccine doses were independently associated with increased risk for hospitalization in our cohort (Figures S3A and S3B).

Adding clade into the model and limiting the dataset to only those patients with sequenced viruses (n = 1,599 patients; Figures S3C and 3C), the results were nearly identical with older age (effector plot in Figure 3D, top), greater number of comorbidities (effector plot in Figure 3D, bottom), male gender, Black or African American race, and lack of vaccination independently associated with increased risk for hospitalization. BMI in this model was borderline significant (p = 0.081). In addition, infection with 20G (adjusted odds ratio [aOR], 0.18; 95% confidence interval [CI], 0.09–0.34), Delta (aOR, 0.28; 95% CI, 0.15–0.52), or Omicron (aOR, 0.45; 95% CI, 0.23–0.90) were all significantly associated with a lowered risk for hospital admission compared with the reference clade, 20A. When we further analyzed all pairwise comparisons of clade within the context of the model, we saw that hospitalization rates in infections with 20G, Delta, and Omicron were significantly different from other clades as well (Figure 3E).

### Viral load is associated with viral clade and vaccination status

**Figure 4:**
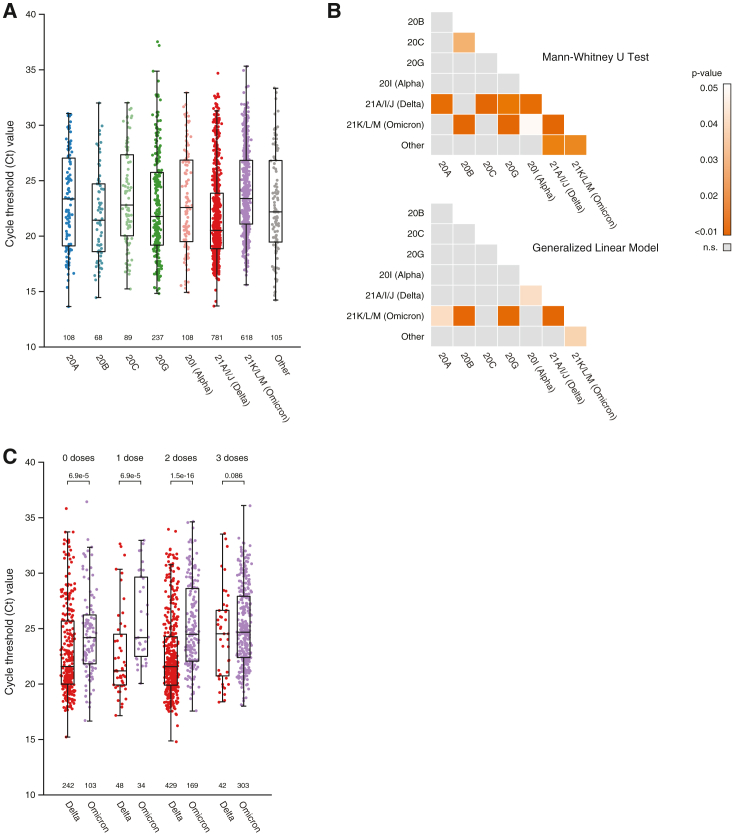
Comparison of SARS-CoV-2 Ct value by clade and vaccination (A) Boxplot of qRT-PCR cycle threshold (Ct) values by clade among sequenced isolates (n = 2,114). Boxplots show the first (Q1), second (median), and third (Q3) quartile, and whiskers represent 1.5 × IQR (defined as Q3−Q1) away from Q1 or Q3. Absolute counts of Ct values per clade are listed below. (B) Heatmaps of p values following pairwise comparison of Ct values between clades. For the top heatmap, a Mann-Whitney U test was used, while for the bottom heatmap, the estimated marginal mean between clades was calculated using a generalized linear model (GLM) controlling for vaccination and hospitalization status. p values in both cases were adjusted using Benjamini-Hochberg (BH) correction to control for false discovery rate. Significant p values (<0.05) shown in orange. (C) Boxplot of Ct values for Delta and Omicron split by vaccination doses before infection. p values were calculated using the Mann-Whitney U test and adjusted using BH correction and are represented above each comparison.

Some studies have suggested an association between viral load in the upper respiratory tract and disease severity,^59^ whereas others have found no association.^60^ Although we had directly comparable qRT-PCR Ct value data on all sequenced specimens as a proxy for viral load, we were unable to include Ct value in the model, as we lacked data on time since symptom onset for most specimens, which is a significant confounder.^61^^,^^62^ However, assuming the time between symptom onset and specimen collection did not vary systematically by clade, we saw that Ct value did vary significantly by clade in a direct comparison (Figures 4A and 4B). Pairwise comparisons using a nonparametric test (Mann-Whitney U test, adjusted p values using Benjamini-Hochberg [BH] correction to control for false discovery rate [FDR]) revealed that most clades significantly differed from Delta (median Ct value, 20.52) and Omicron (median Ct value, 23.42) and that these two clades were significantly different from each other (adjusted p = 4.59e−34; Figure 4B, top).

Apart from time since symptom onset, viral load can be affected by several other factors, including vaccination and the underlying immune status of the host.^63^^,^^64^^,^^65^^,^^66^^,^^67^ To account for this, we modeled Ct value as a dependent variable while controlling for clade, vaccination, and hospitalization. We then calculated the estimated marginal means (EMMs) and adjusted for FDR using BH correction. Even after controlling for these additional confounders, the Ct values of Omicron specimens were significantly higher (lower viral load) than those of 20A, 20B, 20G, Delta, and “other” specimens, while Delta specimens had significantly lower Ct values (higher viral load) than Alpha and Omicron specimens (Figure 4B, bottom). To specifically examine the effects of vaccination on viral load, we compared Ct values of Delta and Omicron specimens as a function of vaccine doses before infection (Figure 4C). Delta specimens had significantly lower Ct values than Omicron among individuals having 0, 1, or 2 vaccine doses prior to infection (Mann-Whitney U test, adjusted p values using BH correction to control for FDR, adjusted p = 6.89e−05, 6.89e−05, and 1.54e−16, respectively), but no significant difference was observed among vaccinated and boosted individuals (3 doses or more; adjusted p = 0.086), similar to previous reports.^65^ Together, this suggests that specific clades are associated with significantly higher or lower viral loads in the upper respiratory tract; differences in time to symptom onset between clades would need to be assessed to validate this observation.

### Viral clade is not associated with outcome, disease severity, or clinical lab values among hospitalized patients

Observing that 20G, Delta, and Omicron were associated with less risk for hospitalization (Figure 3C), we hypothesized that hospitalized patients with those clades would likewise have less severe COVID-19 disease and better outcomes. We tested this in three ways: (1) by building a regression model of hospitalized patient outcomes, (2) by comparing clinical composite scores of patient deterioration between clades, and (3) by examining clinical lab values previously associated with severe disease by clade.

First, we used a multivariable logistic regression to model clinical outcome among hospitalized patients (defined here as no ICU admission versus ICU admission and/or death) while controlling for the previous confounders (n = 1,525 patients; Figures S4A and S4B). Because of the small number of samples for the “Asian” (n = 53) race category in this subset of data, we collapsed them into the “other” category for this model. Similar to the model for risk for hospital admission in Figures S3A and S3B, this model confirmed previous reports that male gender (aOR, 1.84; 95% CI, 1.48–2.29), older age (aOR, 1.02; 95% CI, 1.01–1.02), higher BMI (aOR, 1.02; 95% CI, 1.01–1.03), more comorbidities (aOR, 1.10; 95% CI, 1.04–1.17), and fewer vaccine doses (aOR, 0.70; 95% CI, 0.63–0.78) were independently associated with worse outcomes in hospitalized settings. After adding clade into the model and limiting the dataset to only those patients with sequenced virus isolates (n = 315 patients; Figures S4C and 5A), the results yielded only two significant predictors of ICU admission or death: older age and lack of vaccination. No clades were identified as significant predictors of ICU admission or death. Multiple pairwise comparisons between clades using EMMs and adjusting p values for FDR using BH correction likewise yielded no significance between any clades (p > 0.05; data not shown). Although this may not be unexpected given the somewhat similar distribution of these outcomes between clades (Figure 3B), this latter model failed to identify several other well-characterized predictors of worse clinical outcomes among hospitalized patients (i.e., comorbidities), suggesting that it may be underpowered.

**Figure 5:**
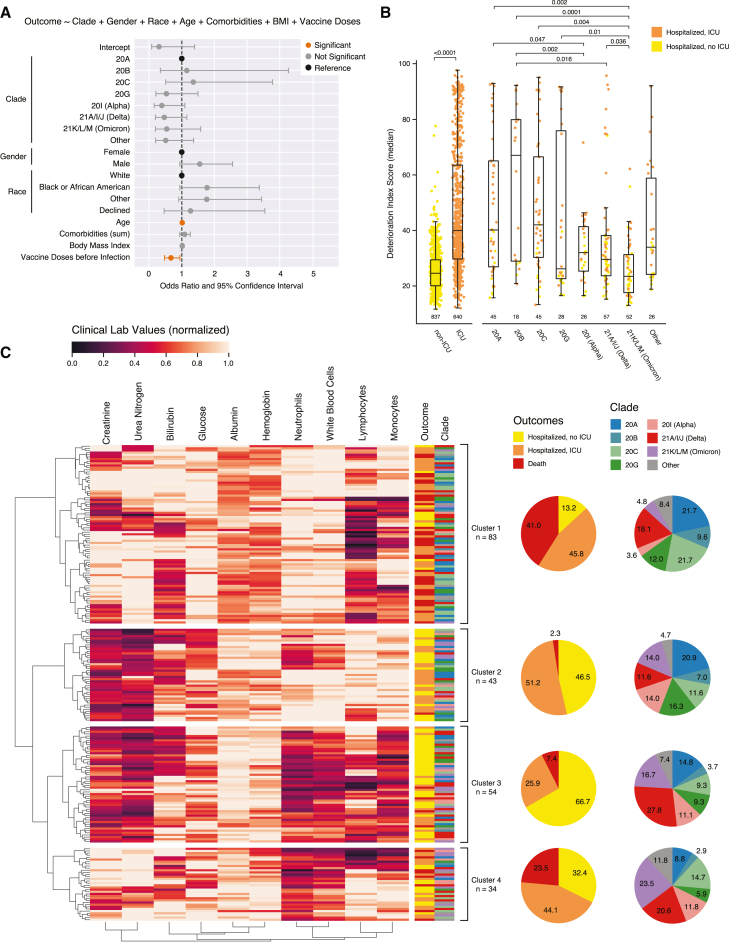
SARS-CoV-2 clade is not associated with outcomes among hospitalized patients (A) Odds ratio plot with 95% confidence interval (CI) as calculated by a multivariable logistic regression modeling ICU admission vs. non-ICU admission within the hospitalized patient cohort (n = 315). Significant features (p < 0.05) are highlighted in orange. Pairwise comparison revealed no significance between clades (not shown). Model parameters are shown in Figure S4. (B) Boxplot of median deterioration index (DI) scores between hospitalized patients comparing patients by ICU admission (left) and clade (right). Data from patients admitted to the ICU are shown in orange, and non-ICU patient data are shown in yellow. Boxplots show the first (Q1), second (median), and third (Q3) quartile, and whiskers represent 1.5 × IQR (defined as Q3−Q1) away from Q1 or Q3. The p value for the ICU vs. non-ICU comparison was calculated using a Mann-Whitney U test. Adjusted p values (Benjamini-Hochberg correction to control for false discovery rate) for clade comparisons were calculated following multiple pairwise comparison for estimated marginal means (represented above bars). (C) Heatmap of average laboratory measurements for all hospitalized patients with sequencing information (n = 214). Clustering was performed using hierarchical clustering with ward linkage. Pie charts represent the distribution of clade and clinical outcome (hospitalized non-ICU, hospitalized ICU, and death) for each cluster. See also Figure S4.

As a second approach, we next compared the median deterioration index (DI) score across clades (Figure 5B). The DI score is a proprietary measure of disease severity (0 as least severe and 100 as most severe) implemented in the EPIC electronic health record system that is automatically calculated for all inpatients daily. The DI score has been correlated with patient outcomes across several pathogenic states, including in COVID-19.^68^^,^^69^ As expected, the median DI score over the course of hospitalization for patients who were never admitted to the ICU (yellow) was significantly lower than that of ICU patients (orange; Mann-Whitney U test, p = 4.89e−111). To compare DI scores between patients infected with different clades of SARS-CoV-2, we modeled DI score as a dependent variable while controlling for clade, vaccination, and ICU admission. We then calculated the pairwise EMM between clades and adjusted p values using BH correction to control for FDR. When controlling for these potential confounders, patients with Omicron had significantly lower DI scores among hospitalized patients in comparison with every clade except Alpha, which itself was significantly different than 20A and 20B (Figure 5B). Although these data are consistent with prior reports indicating that Omicron causes less severe disease and less mortality,^40^ neither 20G nor Delta appears less severe by this metric, despite their associations with lower hospitalization rates (Figure 3C).

Although the DI score is a useful metric for tracking a patient’s deterioration or recovery over time, the algorithm used to calculate it is proprietary and the clinical and demographic data it relies on are unknown. In contrast, several prior studies have shown significant associations between common clinical laboratory measurements and disease severity, including reduced lymphocyte count (i.e., lymphopenia) and elevated monocyte and neutrophil counts.^70^^,^^71^^,^^72^^,^^73^^,^^74^^,^^75^^,^^76^ To assess for clinical lab signatures that may have been associated with one or more infecting clades, we used an unsupervised clustering approach. Briefly, for each hospitalized patient with viral sequence data and complete lab values data (i.e., at least one documented measurement of blood urea nitrogen, creatinine, glucose, bilirubin, albumin, hemoglobin, total white blood cells, neutrophils, monocytes, and lymphocytes during their hospital stay; n = 214 patients), we took the average value for each laboratory measurement over the course of their hospital stay and normalized it on a scale of 0–1. To facilitate visualization and minimize skewing, all values in the upper quartile were capped at the 0.75 quantile value. We then performed hierarchical clustering with ward linkage; an optimal number of clusters was determined to be 4 on the basis of the elbow method using the Silhouette score. Clinical outcomes and viral clade for patients in each cluster are annotated to the right of the heatmap with frequencies depicted in the adjacent pie charts (Figure 5C).

Although the clusters had no strong affiliation with any particular clade, clusters did align well with outcome groups. Cluster 1 comprised the largest group (n = 83) and had the second largest proportion of ICU admissions (45.8%) and the largest proportion of deaths (41%). This group was characterized by higher urea nitrogen, glucose, total white blood cell counts, and neutrophil counts and by lower albumin, hemoglobin, and lymphocyte counts. Cluster 2 had the lowest frequency of death (2.3%) but the highest frequency of ICU admission (51.2%) and was defined by lower creatinine, urea nitrogen, bilirubin, and glucose and by higher lymphocyte and monocyte counts. Cluster 3 was the second largest cluster (n = 54) and had the highest percentage of non-ICU patients (66.7%). This cluster was marked by lower creatinine, urea nitrogen, bilirubin, and glucose (like cluster 2) but also by lower total white blood cell, neutrophil, lymphocyte, and monocyte counts and by higher albumin and hemoglobin levels. Last, cluster 4 had the second highest combined frequency of death and ICU (67.6%), behind only cluster 1. Cluster 4 was slightly less defined and appeared to have a few subclusters. For example, one subcluster of cluster 4 was similar to cluster 3, but with high creatinine and urea nitrogen levels and appeared to be associated with more severe outcomes. Notably, most of the early clades (20A, 20B, 20C, and 20G) were overrepresented in the first two clusters, whereas the opposite was observed for the later clades. However, the clusters overall were more highly correlated with clinical outcome than with clade (Figure 5C). Altogether, these data did not suggest that clades 20G and Delta were associated with less severe disease and better outcomes among hospitalized patients, though Omicron was found to be associated with significantly lower DI scores, suggestive of lower overall disease severity.

### Epidemiological metrics account for the association between clade and hospitalization

Although SARS-CoV-2 clade was independently associated with hospitalization after controlling for demographic and clinical confounders, it was not clearly associated with disease severity or worse outcomes among hospitalized patients (with the exception of Omicron). One potential explanation for this discrepancy might be changes in population-level health determinants, including case count, testing platforms, and surveillance protocols. For example, undercounting of total case counts because of underreporting of at-home tests or asymptomatic infection could result in bias where cases that do present through hospital-affiliated testing centers appear more severe.^77^^,^^78^^,^^79^^,^^80^^,^^81^ Alternatively, increased testing volume prior to seasonal travel could result in cases appearing less severe overall because of the capture of populations with asymptomatic or mild disease that were otherwise unlikely to be tested.^82^^,^^83^^,^^84^

**Figure 6:**
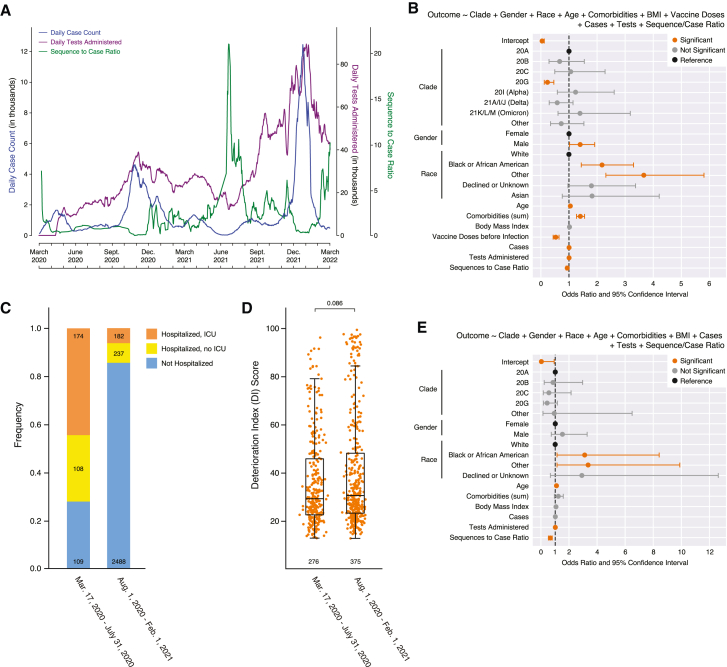
Shifting public health and clinical practices account for the association between clade and risk for hospitalization (A) Line graph of the 7-day rolling average of cases, tests administered, and sequence-to-case ratio for Cook County. Number of sequences on the basis of data in this study. (B) Odds ratio plot with 95% confidence intervals (CIs) as calculated by a multivariable logistic regression modeling risk for hospitalization with epidemiological features (n = 1,597). Significant features (p < 0.05) are highlighted in orange. (C) Relative frequency of hospital admission and ICU admission in this dataset from March 17, 2020, to July 31, 2020, and from August 1, 2020, to February 1, 2021. Absolute counts listed in respective bars. (D) Boxplot of DI score at hospital admission for all inpatients in this dataset from March 17, 2020, to July 31, 2020, and from August 1, 2020, to February 1, 2021. Boxplot shows the first (Q1), second (median), and third (Q3) quartile, and whiskers represent 1.5 × IQR (defined as Q3−Q1) away from Q1 or Q3. p value calculated using Mann-Whitney U test. (E) Odds ratio plot with 95% confidence intervals (CIs) as calculated by a multivariable logistic regression modeling risk for hospitalization with epidemiological features, limited using only data from August 1, 2020, to February 1, 2021 (n = 285). Significant features (p < 0.05) are highlighted in orange. Pairwise comparison revealed no significance between clades (not shown). Model parameters for (B) and (E) are shown in Figure S5.

Although several known confounders were controlled for in the original model (i.e., individualized demographic and clinical features; Figure 3C), population-level epidemiological parameters such as tests administered, number of cases, and surveillance sampling rates were not accounted for and may be a source of uncaptured bias (Figure 6A). For example, the sequence-to-case ratio (i.e., the proportion of normalized daily sequenced specimens to normalized daily reported COVID-19 cases) can be used to track surveillance rates over time; if the amount of sequencing being performed is proportional to the number of contemporaneously reported COVID-19 cases, then this ratio should be roughly 1. However, this metric indicated undersampling of sequences during late 2020 (potentially of 20A, 20B, 20C, and 20G) and oversampling in the latter half of 2021 (likely of Delta). When these metrics (case count, tests administered, and sequence-to-case ratio) were added to our model predicting risk for hospitalization (n = 1,597 patients; IDPH did not record data on March 23, 2020, so two samples corresponding to that date were dropped; Figures 6B and S5A), all three metrics were shown to be significant predictors. Likewise, all previous significant predictors are retained with the exception of Delta and Omicron, which lost significance. 20G remained a significant predictor of lack of hospitalization, which was confirmed through multiple pairwise testing that showed a statistically significant difference between 20G and all other clades (data not shown, FDR < 0.05).

Clade 20G became regionally prevalent in the United States in late 2020. It shared the same Spike protein sequence as clades 20A, 20B, and 20C with a single point mutation in this gene relative to the Wuhan reference sequence (Spike:D614G). Although changes in other viral proteins have been linked to differences in viral load and the host response,^85^ there is no clear biological explanation for clade 20G’s decreased risk for hospitalization after controlling for the clinical, demographic, and epidemiological variables above. However, an additional possible confounding factor for the risk of hospitalization is the evolution of the standard of care (SOC) for management of COVID-19 patients over the course of the pandemic. Over the first year of the pandemic, the SOC for COVID-19 patients changed rapidly as clinicians became more familiar with the anticipated disease course; identified risk factors for severe disease and the need for hospitalization/ICU care; and codified successful therapeutic approaches such as ventilator management, steroid treatments, or antiviral therapeutics into their practice.

At NMH and affiliated clinics, the SOC for COVID-19 patients changed rapidly over the first six months of the pandemic, and we estimate that it had become generally standardized by August 1, 2020. Comparing hospitalization from the start of our study on March 17, 2020, through July 31, 2020, with the six months immediately following, we saw a dramatic decline in the relative frequency of hospital admissions and ICU admissions (Figure 6C). However, when we compared the DI score at hospital admission, we observed no statistically significant change over these time frames, suggesting that underlying disease severity stayed relatively constant (Figure 6D). Additionally, early in the COVID-19 pandemic, SARS-CoV-2 testing in Chicago was limited largely to those with high-risk exposure, travel from countries with high case rates, or severe disease. This limitation and bias in testing could also have artifactually increased the observed proportion of hospitalized vs. non-hospitalized patients until broader outpatient testing became available. This suggests that changes in the SOC and/or testing availability, not disease severity, were responsible for the observed increased frequency of hospital admission early in the pandemic.

To account for changes in SOC, we reran our previous hospitalization model including epidemiological metrics (Figure 6B), but limited the dataset from August 1, 2020, to February 1, 2021, when the SOC was relatively stable and 20G was predominant. Because of the low sample numbers, we collapsed “Alpha” (n = 1) into the “other” clade category and “Asian” (n = 12) into the “other” race category. We also removed the vaccination feature because of the low number of fully vaccinated individuals during this time frame (n = 2). In this model, clade was not a significant predictor of hospitalization (n = 285 patients; Figures 6E and S5B). Multiple pairwise comparisons also resulted in no significance between any two clades (data not shown). Age, race (Black or African American and “other”) and epidemiological features (tests administered and the sequence-to-case ratio) remained significant. To validate these results with a comparable external dataset, we sequenced samples collected from 249 patients presenting to hospitals and clinics in the Chicago suburb-based Northshore University HealthSystem between January 2021 and March 2021. Because of the low numbers of individual clades in this dataset, we broadly collapsed clades into “VOC” (n = 75), 20G (n = 102), and “other” (n = 72). Similar to the results in our larger Northwestern University dataset, a model of hospitalization as the primary outcome while controlling for clade, gender, age, BMI, number of symptoms and infectivity (calculated as number of daily cases divided by number of daily tests administered) detected no significant differences between clades, nor did multiple pairwise comparison between 20G, the “VOC” group, and the “other” group (data not shown). Taken together, these results suggest that, with the exception of the Omicron VOC as demonstrated by DI score, SARS-CoV-2 clade is not a significant predictor of patient outcome or disease severity after controlling for demographic, clinical, epidemiological, and SOC confounders. Furthermore, failure to control for these population-level confounders may result in spurious conclusions that muddle our understanding of variants, disease severity, and perceived risk.

## Discussion

In this study, we report the molecular epidemiology of the COVID-19 pandemic over a two-year period in Cook County, Illinois, and assess the association between viral clade and clinical outcome. We initially found that although clade 20G, the Delta VOC, and the Omicron VOC were significantly associated with a lower risk for hospitalization when controlling for individualized clinical and demographic confounders (Figure 3C), they were not associated with ICU admission among hospitalized patients, and only the Omicron VOC showed significantly lower disease severity as measured by DI score (Figures 5A and 5B). Further analyses that controlled for population-level epidemiological factors, including case counts, testing, and sampling, negated the association of Delta and Omicron with hospitalization (Figure 6B). Limiting the time frame of the model to account for changing clinical SOC negated the association of 20G with hospitalization (Figure 6E). These results demonstrate the importance of non-virological (i.e., clinical, demographic, and epidemiological) data at both the individual and population levels when trying to model the effect of viral mutation on disease severity.

Several prior efforts have been made to assess the association between SARS-CoV-2 variants with disease severity. Reports by Trobajo-Sanmartín et al.^86^ and Funk et al.^87^ found that the Alpha VOC showed an increased risk for hospitalization and a higher odds of ICU admission relative to non-VOCs, respectively. Veneti et al.^88^ found that the Beta VOC also showed an increased risk for hospitalization as well as ICU admission compared with non-VOCs. Nonaka et al.^89^ showed that a significant increase in ICU admission among young patients with no comorbidities shortly after the introduction of the Gamma VOC, suggesting its increased severity. In a meta-analysis, Lin et al.^90^ found that VOCs had an increased risk for hospitalization, ICU admission, and mortality compared with earlier clades with Delta accounting for the highest risk for ICU admission and mortality. Despite each studies’ strengths, they each control for only a limited subset of potentially confounding variables depending on the available metadata. For example, the aORs reported by Funk et al.^87^ are adjusted only for gender, age, week of reporting, and country. With different studies accounting for a wide range of confounders, even meta-analyses can be skewed depending on how studies are weighted.

As we demonstrated, after controlling for an expanded set of population-level confounders, apparent associations could be explained by changes in case reporting, testing, sampling, and clinical care practices. The inclusion of epidemiological factors and the identification of the shifting SOC points to the complexity in which we should aim to understand disease severity. Features not directly reported with the patient, such as the number of tests administered, and sequencing strategies may unintentionally bias our understanding of results. Most models emphasize features that represent individualized biological significance, such as demographics and clinical data, to model disease severity. However, recent findings have shown that non-biological factors have large effects on disease severity; including how the study was performed.^91^ Advanced models should strive to use an integrated analysis approach, combining data from all sources to minimize confounding factors. Models that account for more variables across a bigger population, but over a limited time frame, may be the most valuable in comparing disease severity. Notably, historical comparison of variants that did not co-circulate in the same population at the same time may lead to spurious results and are challenging to properly control in these types of study.

Our results confirmed several known predictors of disease severity and clinical outcome, including demographic factors (age, gender, race) and pre-existing conditions (BMI, comorbidities) (Figures 3C, 5A, 6B, and 6E).^53^^,^^54^^,^^55^^,^^56^^,^^57^ Vaccine doses prior to infection was likewise strongly associated with better outcomes in every model, confirming many previous studies (Figures 3C, 5A, 6B, and 6E).^25^^,^^26^^,^^27^^,^^28^ Our analysis also confirmed the results of previous studies that found associations with more severe disease outcomes and reduced lymphocyte count, elevated neutrophil count, and decreased albumin and hemoglobin (Figure 5C).^73^^,^^92^^,^^93^^,^^94^^,^^95^^,^^96^^,^^97^^,^^98^ Although certain clinical lab value signatures were more clearly associated with disease severity, no signatures were clearly associated with any given clade, again suggesting that clade is poorly associated with severity or clinical outcome. A study conducted by Lazar et al.^99^ showed that lower lymphocytes, hemoglobin, and albumin and higher neutrophil-to-lymphocyte ratio were correlated with mortality. These findings were confirmed by Moutchia et al.^100^^,^^101^ and Ziuzia-Januszewska et al^100^^,^^101^ Interestingly, Ziuzia-Januszewska et al. also found that there was no significant difference in mortality between the second and third wave, thereby concluding that Alpha had no effect on disease severity, which was also confirmed by our results.

These data also confirmed several recent reports on the Omicron VOC, including its rapid spread (Figure 2D),^102^ lower disease severity compared with Delta (Figure 5B),^103^^,^^104^ and lower viral load compared with Delta,^65^ even after controlling for vaccination status (Figures 4A and 4C). The lower viral load in the upper airway at the time of sampling appears counterintuitive to its enhanced transmissibility, especially given that the higher transmissibility of previous VOCs was often attributed to higher viral loads.^61^^,^^105^ Recent studies profiling the mutations of the Omicron Spike protein have suggested that it can bind more strongly to ACE2, and that it prefers to enter cells via an alternate endosomal pathway.^102^^,^^106^^,^^107^ These data suggest that the Omicron VOC may have a slightly different tropism and prefer to replicate in the upper airway, which might explain both the increased transmissibility and decreased disease severity.

In sum, this study characterized the molecular epidemiology of the COVID-19 Chicago pandemic over a two-year time frame and found no association between viral clade and disease severity or clinical outcome after controlling for demographic, clinical, and epidemiological confounders. The one exception to this was the Omicron VOC, which showed evidence of decreased severity among hospitalized patients when comparing composite scores of clinical deterioration. Although there are some ongoing genomic surveillance efforts,^108^^,^^109^^,^^110^ this study highlights the regional heterogeneity of the COVID-19 pandemic, and points to the importance of continued, widespread surveillance both within the United States and around the globe.^111^ Understanding how viral evolution is associated with transmissibility or clinical severity is important to inform clinical care, inform the public health response, and to develop novel therapeutics and vaccines that are forward-looking to future variants. Finally, this work further emphasizes the need for the integration of epidemiological factors with individualized datasets to control for population-level confounders that might not otherwise be captured in health records alone.

### Limitations of the study

Despite its strengths, this study has several limitations. First, this study is a single-center cohort study which may not reflect the true effects of the general population. Although our catchment area spanned the greater Chicago area, the specimens were biased to the area around NMH, which was reflected in the slight demographic skew relative to the city (Table S1). That being said, our comparisons of the sequence data to greater Illinois and the United States suggested a representative sampling (Figure S2), while the models run without clade information largely recapitulated expected results (Figures S3 and S4). More specimens from additional sites would greatly improve the power of the models; we suspect that lack of power in our model of hospitalized patients is the reason that Omicron was not associated with decreased severity (Figure 5A). One feature our study did not control for was the possibility that patients had developed natural immunity from prior infection. Risk for hospitalization for those who had recent previous infections are relatively low and thus may confound with our understanding of severity.^112^ Although this is hard to control for, it again suggests that study periods should be limited to catch similarities at the population level.

## STAR★Methods

### Key resources table

**Table:** 

REAGENT or RESOURCE	SOURCE	IDENTIFIER
**Biological samples**		

Nasal swabs in viral transport media	SARS-COV-2 biorespository at the Center for Pathogen Genomics and Microbial Evolution at Northwestern University	2,114 unique identifiers in attached table.

**Critical commercial assays**		

QIAmp Viral RNA Mini Kit	Qiagen	52906
QIAamp 96 Virus QIAcube HT Kit	Qiagen	57731
TaqPath 1-Step RT-qPCR Master Mix, CG	Thermo Scientific	A15300
PW384A: plexWell PW384 -- Index Set A	Seqwell	PW384
SuperScript IV Reverse Transcriptase 200 rxns	Thermo Scientific	18090200
MiSeq Reagent Kit v2 (500 cycle)	Illumina	MS-102-2003
Random Hexamers (50 M)	Thermo Scientific	N8080127
RNaseOUT Recombinant Ribonuclease Inhibitor	Thermo Scientific	10777019
dNTP Mix	Thermo Scientific	R0194
Q5 Hot Start High-Fidelity DNA Polymerase - 500 units	NEB	M0493L
PhiX Control v3	Illumina	FC-110-3001
AmpureXP beads	Beckman Coulter	A63881
NEB Ultra II End Prep Enzyme mix	NEB	E7546L
Nanopore Native Barcoding Expansion kits	Oxford Nanopore	EXP-NBD-104, EXPNBD114
NEBNext Ultra II Ligase	NEB	E6056L
Oxford Nanopore Genomic DNA by Ligation kit	Oxford Nanopore	SQK-LSK109
Type R9.4.1 flow cells	Oxford Nanopore	FLO-MIN106D
KAPA HyperPrep Kit	KAPA	KK8500
NEXTflex barcodes	Perkin Elmer	NOVA-514105

**Deposited data**		

SARS-CoV-2 genomes (downloaded)	GISAID	https://www.gisaid.org/
SARS-CoV-2 genomes (uploaded)	GISAID	GISAID Identifier: EPI_SET_230918fy https://doi.org/10.55876/gis8.230918fy
Chicago COVID-19 hospitalizations	Chicago Department of Public Health	https://www.chicago.gov/city/en/depts/cdph.html
Cook County COVID-19 confirmed cases, hospitalizations, deaths and vaccination status	Illinois Department of Public Health	https://dph.illinois.gov/
US COVID-19 confirmed cases, hospitalizations, deaths, vaccination status	Center for Disease Control and Prevention	https://www.cdc.gov/
Illinois confirmed cases, deaths and vaccination	Illinois Department of Public Health	https://dph.illinois.gov/
Illinois confirmed hospitalizations	U.S. Department of Human and Health Services	https://beta.healthdata.gov/

**Oligonucleotides**		

Artic V4.1 NCOV-2019 Panel, 500rxn	IDT	10008554
Hs_RPP30 Positive Control	IDT	10006626
2019-nCoV_N_Positive Control	IDT	10006625
RNase P Reverse Primer Aliquot, 50 nmol	IDT	167485630
RNase P Forward Primer Aliquot, 50 nmol	IDT	167485629
RNase P (ATTO 647) Probe, 25 nmol	IDT	167485625
nCOV_N1 Probe Aliquot, 25 nmol	IDT	167485628
nCOV_N1 Reverse Primer Aliquot, 50 nmol	IDT	167485627
nCOV_N1 Forward Primer Aliquot, 50 nmol	IDT	167485626
ARTIC nCoV-2019 V3 Panel, 500rxn	IDT	10006788

**Software and algorithms**		

Python	Python Software Foundation	version 3.8.8
Pandas (Python package)	NumFOCUS	version 1.1.5
Numpy (Python package)	NumFOCUS	version 1.23.2
collections (Python package)	Python Software Foundation	version 3.8.8
itertools (Python package)	Python Software Foundation	version 3.8.8
datetime (Python package)	Python Software Foundation	version 3.8.8
Scikit-learn (Python package)	Pedregosa et al. (2011)^113^	version 0.24.1
statsmodels (Python package)	Seabold and Perktold (2010)^114^	version 0.12.2
scipy (Python package)	NumFOCUS	version 1.6.2
seaborn (Python package)	Waskom (2021)^115^	version 0.11.1
math (Python package)	Python Software Foundation	version 3.8.8
matplotlib (Python package)	Hunter (2007)^116^	version 3.3.4
R	The R Foundation	version 4.1.1
dplyr (R package)	Wickham et al. (2022)^117^	version 1.0.8
emmeans (R package)	Searle, Speed & Milliken (1979)^118^, package source: https://github.com/rvlenth/emmeans	version 1.8.00
treeio (R package)	Wang et al. (2020)^119^ & Yu et al. (2017)^120^	version 1.18.1
ggtree (R package)	Yu et al. (2017)^120^	version 3.2.1
ape (R package)	Paradis and Schliep (2019)^121^	version 5.6–2
ggtreeExtra (R package)	Yu (2022)^122^ & Xu et al. (2021)^123^	version 1.4.2
ggplot2 (R package)	Wickham (2016)^124^	version 3.3.6
RColorBrewer (R package)	Brewer et al. (2003)^125^	version 1.1–3
MAFFT	Katoh et al. (2013)^126^	version 7.453
MEGAX	Kumar et al. (2018)^127^	version 10.1.8.69
IQ-Tree	Minh et al. (2020)^128^	version 2.0.5
ModelFinder (IQ-Tree function)	Kalyaanamoorthy et al. (2017)^129^	version 2.0.5
TreeTime	Sagulenko et al. (2018)^130^	version 0.7.6

### Resource availability

#### Lead contact

Further information and requests for resources and reagents should be directed to and will be fulfilled by the lead contact, Judd F. Hultquist (judd.hultquist@northwestern.edu).

#### Materials availability

This study did not generate new unique reagents.

#### Data and code availability

##### Data

• Processed next-generation sequencing data have been deposited at GISAID and are publicly available as of the date of publication. Sequence IDs for the sequences used are available at: https://github.com/tedlinghu/molecular_epidemiology_covid19_chicago/tree/main/Data. Resulting phylogenetic trees were visualized using the R packages ggtree v3.2.1 and ggtreeExtra v1.4.2.
• This paper analyzes existing, publicly available data; publicly available sequences were extracted from GISAID database^131^ at https://epicov.org/epi3/epi_set/230801ve?main=true (USA), https://epicov.org/epi3/epi_set/230802kd?main=true (world) and https://epicov.org/epi3/epi_set/230801zh?main=true (Cook County). Accession numbers are listed in the key resources table.
• Public health and epidemiological data were extracted from Illinois Department of Public Health,^46^ Chicago Department of Public Health,^47^ Center for Disease Control^132^ and Health and Human Services.^133^ Hospitalization data before June 13^th^, 2020, is not available from IDPH, so Chicago Department of Public Health (CDPH) data was used for March 17^th^, 2020, to June 13^th^, 2020.
• The individualized clinical data reported in this study cannot be deposited in a public repository due to IRB constraints.

##### Code

All original code has been deposited at Github and is publicly available as of the date of publication. DOIs are listed in the key resources table. https://github.com/tedlinghu/molecular_epidemiology_covid19_chicago/tree/main/Data.

Any additional information required to reanalyze the data reported in this paper is available from the lead contact upon request.

### Experimental model and study participant details

The study did not involve direct interaction with human subjects and obtained de-identified electronic health data on study participants.

### Method details

#### Ethics statement

All handling and extraction of patient data was approved through the Northwestern University Internal Review Board (IRB ID: STU00206850, STU00212260, STU00212267). Personal health information (PHI) was removed prior to analysis and samples were coded with a unique identifier. Unique identifiers were maintained by select study personnel approved by the IRB and stored on a secure, protected server. Data including PHI is stored on a secure server following HIPAA compliance with accessibility limited to study team members approved by the IRB.

#### Clinical data extraction

Early in the COVID-19 pandemic, IRB approval (STU00212267) was obtained to create a data mart of all adults diagnosed and treated for COVID-19 across Northwestern Medicine (NM) using the NM Enterprise Data Warehouse (NMEDW). The NMEDW is a joint initiative across Northwestern University Feinberg School of Medicine and Northwestern Memorial Healthcare Corporation to create a single, comprehensive, and integrated repository of all clinical and research data sources to facilitate research, clinical quality, and healthcare operations. The following electronic data elements were extracted and complied once a week from the NMEDW for all adults diagnosed with COVID-19: demographics, health system visits/level of care (i.e., outpatient, ED, hospital, ICU), vital signs, specific laboratory test results, imaging studies, co-morbid conditions/diagnoses (via ICD9/ICD10 coding), pharmacologic therapy (initially via NMEDW pharmacy/medication records then confirmed as needed with electronic chart review).

#### Sample collection and viral load determination

Residual diagnostic specimens from individuals testing positive for SARS-CoV-2 in the Northwestern Medicine healthcare system were collected as part of an established biobank in the Center for Pathogen Genomics and Microbial Evolution at the Northwestern University Feinberg School of Medicine. Samples collected between March 17th, 2020, through March 17th, 2022, were included as part of this study (STU00206850, STU00212260).

Viral RNA was extracted from nasopharyngeal specimens stored in viral transport media (VTM) utilizing the QIAamp Viral RNA Minikit and the QIAamp 96 Virus QIAcube HT Kit (Qiagen); VTM only controls were included in each extraction. Laboratory testing for the presence of SARS-CoV-2 was performed by quantitative reverse transcription and PCR (qRT-PCR) with the CDC 2019-nCoV RT-PCR Diagnostic Panel utilizing N1 and RNase P probes as previously described [https://www.cdc.gov/coronavirus/2019-ncov/lab/rt-pcr-panel-primer-probes.html]. Positive and negative controls for SARS-CoV-2 and RNase P were included in each qRT-PCR experiment alongside the VTM only sample from the RNA extraction, a no template control, and standard curves for SARS-CoV-2 and RNase P. Specimens with RNase P cycle thresholds (Ct) above 35 were of insufficient quality and were excluded from future studies. N1 Ct values less than or equal to 35 were considered positive, these Ct values were used in all subsequent analyses.

#### cDNA synthesis and viral genome amplification

cDNA synthesis was performed with SuperScript IV First Strand Synthesis Kit (Thermo) using random hexamer primers according to manufacturer’s specifications. Direct amplification of the viral genome cDNA was performed in multiplexed PCR reactions to generate ∼400 base pair amplicons tiled across the genome. The multiplex primer set, comprised of two non-overlapping primer pools, was created using Primal Scheme and provided by the Artic Network (versions 3, 4, and 4.1 releases) [https://www.protocols.io/view/ncov-2019-sequencing-protocol-bp2l6n26rgqe/v1]. PCR amplification was carried out using Q5 Hot Start HF Taq Polymerase (NEB) with 5 μL of cDNA in a 25 μL reaction volume. A two-step PCR program was used with an initial step of 98°C for 30 s, then 35 cycles of 98°C for 15 s followed by 5 min at 65°C. Separate reactions were carried out for each primer pool and validated by agarose gel electrophoresis.

#### Sequencing library preparation and whole genome sequencing

Three sequencing library preparation protocols were utilized over the course of this study; all protocols made use of one of the ARTIC amplicon pools (v3, v4, or v4.1). The sequencing library preparation approach for the Oxford Nanopore MinION was adapted from the ARTIC Network protocol and Oxford Nanopore protocol “PCR tiling of COVID-19 virus”.^,^^134^ Amplicons from both primer pools were combined and purified with a 1x volume of AmpureXP beads (Beckman Coulter). A total of 50 ng of DNA was treated with NEB Ultra II End Prep Enzyme mix (NEB). Up to 24 specimen libraries were barcoded using Nanopore Native Barcoding Expansion kits (Oxford Nanopore) and NEBNext Ultra II Ligase (NEB) for simultaneous sequencing. Uniquely barcoded samples were pooled and cleaned with a 0.4x volume of AmpureXP beads. Adapter ligation and cleanup was performed using the Oxford Nanopore Genomic DNA by Ligation kit (Oxford Nanopore) according to manufacturer specifications. Libraries were sequenced on the Nanopore MinION device using FLO-MIN106D Type R9.4.1 flow cells. This sequencing approach was the primary method used from March 2020 to August 2020 and was exclusively used with libraries generated with the ARTIC v3 primer set.

This study used two separate approaches to library preparation for sequencing on the Illumina MiSeq platform. The first sequencing library approach was adapted from previously published methods.^49^ Briefly, amplicons from both primer pools were combined and purified with a 1x volume of AmpureXP beads (Beckman Coulter). A total of 75 ng of DNA was treated with KAPA HyperPrep End Prep Enzyme mix (KAPA). Up to 96 specimen libraries were barcoded using NEXTflex barcodes and KAPA HyperPrep DNA Ligase (KAPA) for simultaneous sequencing. Uniquely barcoded samples were pooled and purified with a 0.8x volume of AmpureXP beads. Library amplification was performed using KAPA HiFi HotStart with KAPA Library Amp Primers. Amplicons were purified with a 0.8x volume of AmpureXP beads and normalized to 5 nM and pooled. The pooled library was denatured and loaded onto a MiSeq v2 500 cycle kit (Illumina). This method was primarily used from September 2020 to November 2020 and was used exclusively with libraries generated with the ARTIC v3 primer set. The second protocol for sequencing library preparation of genome amplicon pools was performed using the SeqWell plexWell 384 kit per manufacturer’s instructions and was the primary method used from December 2020 through March 2022. Pooled libraries were sequenced on the Illumina MiSeq using the V2 500 cycle kit.

Viral genome consensus sequences were determined from sequencing reads as previously described.^135^ Sequencing reads were trimmed to remove adapters and low-quality sequences using Trimmomatic v0.36^136^. Trimmed reads were aligned to the reference genome sequence of SARS-CoV-2 (accession MN908947.3) using bwa v0.7.15^137^. Pileups were generated from the alignment using samtools v1.9^138^ and consensus sequence determined using iVar v1.2.2^135^ with a minimum depth of 10, a minimum base quality score of 20, and a consensus frequency threshold of 0 (i.e., majority base as the consensus). To validate our sequencing results and rule out potential contamination, 5% of all samples were randomly selected for repeat sequencing, including an independent RNA extraction, cDNA synthesis, multiplex PCR amplification, library preparation, and Illumina sequencing run. All repeat sequencing results for this study resulted in identical lineage designations.

Between March 17th, 2020, and March 17th, 2022, a total of 3,131 samples were sequenced from Cook County. Out of 3,131 generated consensus sequences, 259 failed to meet our coverage thresholds (>90% coverage with at least 10 reads) and were excluded from further analysis. A majority of failed sequences were derived from specimens with low viral loads, corresponding to a cycle threshold (Ct) value greater than 29; sequencing failure rates for specimens with low viral loads were consistent across time and variants analyzed. Of the 2,872 remaining sequences, 163 sequences were found to come from a patient already in the dataset and 595 were from children less than 18 years of age. These were excluded for a final number of 2,114 sequences. Patient demographics (Table S1) and lineage designations (Figure S2) were compared for these isolates to ensure they were representative of our larger cohort and population.

### Quantification and statistical analysis

#### Statistical analysis

All statistical analyses were done in Python (v3.8.8) using Pandas (v1.1.5), Numpy (v1.23.2), statsmodels (v0.12.2), scipy (v1.6.2) packages and R (v4.1.1) using dplyr (v1.0.8) and emmeans (v1.8.00). Clinical outcome and ICU admission were modeled using a multivariable logistic regression model by applying the logit function in statsmodels.^114^ To compare all pairwise differences in each model, we calculate the estimated marginal means (EMM) using both Python and R.^118^ For Python, we used the get_margeff(at = ‘mean’) function after generating the model, setting each clade as the reference, and then correcting the p values using multipletests(alpha = 0.05, method = ‘fdr_bh’). For R, we used the emmeans package in R to control for clinical outcome and vaccination status. The different uses were for ease of data manipulation within each code language. All graphs were generated in matplotlib^116^ (v3.3.4) and seaborn^115^ (v0.11.1). The clustermap was generated using the clustermap function from seaborn with “ward” linkage. This generates hierarchical cluters both row and columns wise.^139^ The optimal number of clusters was determined by the highest silhouette score between 4 and 10 clusters^140^ from the Scikit-learn package.^113^

#### Phylogenetic analysis

We used Nextclade (https://clades.nextstrain.org/) to classify viral clades. Genome sequences were aligned using MAFFT v7.453 software^126^ and performed a visual inspection using MEGAX v10.1.8 69 to assess and correct misaligned blocks due to the misplaced gaps.^127^ All Maximum Likelihood (ML) phylogenies were inferred with IQ-Tree v2.0.5^128^ using its ModelFinder function^129^ before each analysis to estimate the nucleotide substitution model best-fitted for each dataset by means of Bayesian information criterion (BIC). For every phylogeny, SARS-CoV-2 reference genome Wuhan-Hu-1 (NC_045512) was included to facilitate tree rooting. We assessed the tree topology for each phylogeny with the Shimodaira–Hasegawa approximate likelihood-ratio test (SH-aLRT)^141^ with 1000 replicates, also adding ultrafast bootstrap (UFboot)^142^ for the Chicago phylogeny. TreeTime v0.7.6^130^ was used for the assessment of root-to-tip correlation, the estimation of time scaled phylogenies and ancestral reconstruction of most likely sequences of internal nodes of the tree and transitions between geographical locations along branches (the latter only for USA and global phylogenies). TreeTime was run using an autocorrelated molecular clock under a skyline coalescent tree prior, setting a clock rate of 8x10^−4^ and a clock rate standard deviation of 4x10^−4^ to match the widely used Nextstrain pipeline (Nextstrain.org) and as confirmed in our previous studies.^67^ We used the sampling dates of the sequences to estimate the evolutionary rates and determine the best rooting of the tree using root-to-tip regression with least-squares method. Due to the high number of sequences available for the USA ML phylogeny, we performed a temporal subsampling of all the sequences available from the USA in GISAID, randomly sampling 100 sequences per month since the beginning of the epidemic until April 1^st^, 2022 (n = 2900 sequences). For the global analysis we downloaded the curated GISAID global alignment on July 18^th^, 2022, and removed all sequences obtained after April 1^st^, 2022 (n = 1861 sequences). Both datasets were merged with the sequences generated for this study to perform the corresponding analyses. Sequence IDs for the sequences used are available at: https://github.com/tedlinghu/molecular_epidemiology_covid19_chicago/tree/main/Data. Resulting phylogenetic trees were visualized using the R packages ggtree v3.2.1 and ggtreeExtra v1.4.2 and also at https://epicov.org/epi3/epi_set/230801ve?main=true (USA), https://epicov.org/epi3/epi_set/230802kd?main=true (world), and https://epicov.org/epi3/epi_set/230801zh?main=true (Cook County).

## Supplemental information

Document S1. Figures S1–S5

Table S2. GISAID accession ID numbers for deposited sequences, related to Figures 2–6

Table S3. A lookup table that maps Pangolin lineage to Nextstrain clade, related to Figures 2–6

Document S2. Article plus supplemental information
