# Supplementary material for: Integration of individualized and population-level molecular epidemiology data to model COVID-19 outcomes
Source: Cell Rep Med. 2024 Jan 16;5(1):101361. doi: 10.1016/j.xcrm.2023.101361 (PMC10829796; doi:10.1016/j.xcrm.2023.101361)
Supplement: Document S1. Figures S1–S5 [file mmc1.pdf]

Cell Reports Medicine, Volume *5*

Supplemental information

Integration of individualized and population-level molecular epidemiology data to model COVID-19 outcomes

Ted Ling-Hu, Lacy M. Simons, Taylor J. Dean, Estefany Rios-Guzman, Matthew T. Caputo, Arghavan Alisoltani, Chao Qi, Michael Malczynski, Timothy Blanke, Lawrence J. Jennings, Michael G. Ison, Chad J. Achenbach, Paige M. Larkin, Karen L. Kaul, Ramon Lorenzo-Redondo, Egon A. Ozer, and Judd F. Hultquist

SUPPLEMENTARY FIGURES

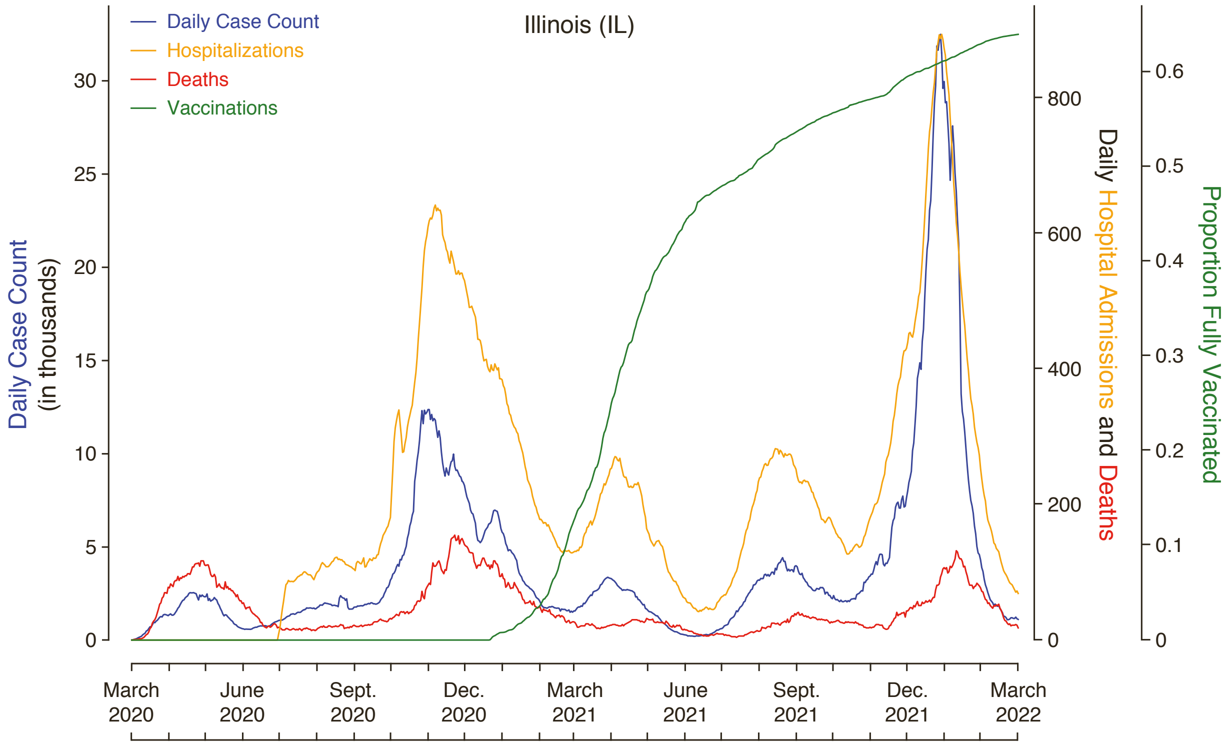

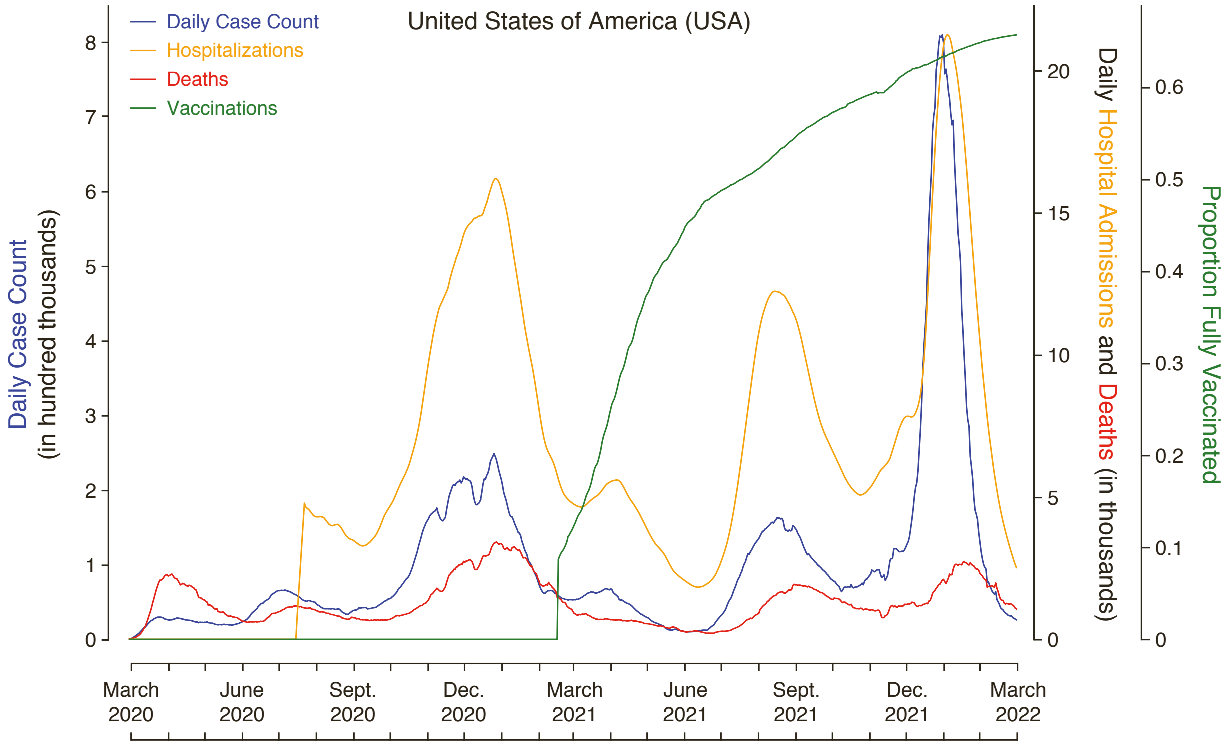

Figure S1: Summary of the COVID-19 pandemic in the state of Illinois and the United States of America. Epidemiology of COVID-19 as represented by 7-day rolling average of cases (blue), hospitalizations (orange) and deaths(red) between March 17th, 2020 to March 17th, 2022. The cumulative fully vaccinated (1 dose of Johnson & Johnson or two doses of other vaccines) proportion of the population is shown in green. Related to Figure 1.

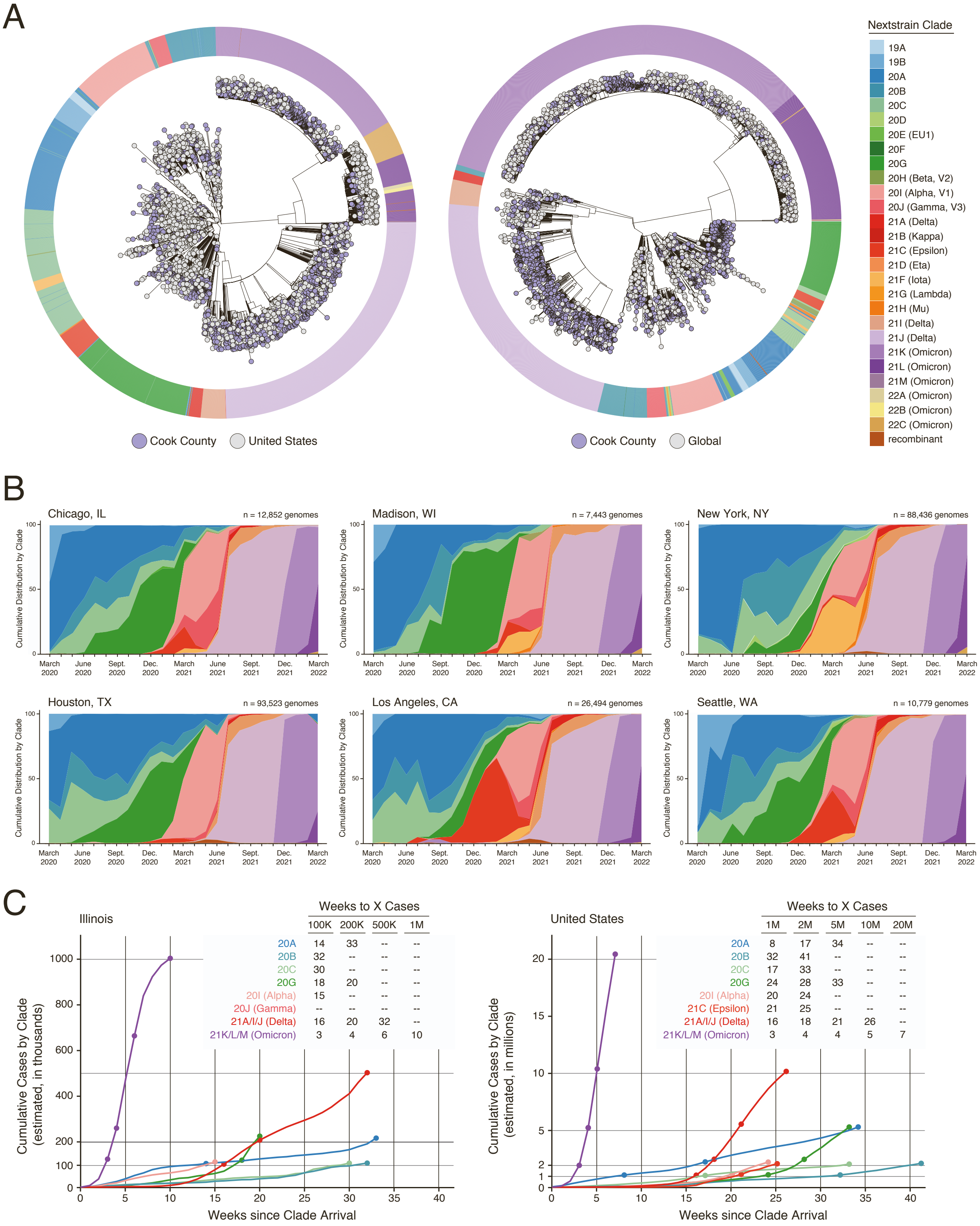
A
Nextstrain Clade
19A
19B
20A
20B
20C
20D
20E (EU1)
20F
20G
20H (Beta, V2)
20I (Alpha, V1)
20J (Gamma, V3)
21A (Delta)
21B (Kappa)
21C (Epsilon)
21D (Eta)
21F (Iota)
21G (Lambda)
21H (Mu)
21I (Delta)
21J (Delta)
21K (Omicron)
21L (Omicron)
21M (Omicron)
22A (Omicron)
22B (Omicron)
22C (Omicron)
recombinant
Cook County
United States
Cook County
Global
B
Chicago, IL
n = 12,852 genomes
Madison, WI
n = 7,443 genomes
New York, NY
n = 88,436 genomes
Houston, TX
n = 93,523 genomes
Los Angeles, CA
n = 26,494 genomes
Seattle, WA
n = 10,779 genomes
Cumulative Distribution by Clade
March 2020
June 2020
Sept. 2020
Dec. 2020
March 2021
June 2021
Sept. 2021
Dec. 2021
March 2022
C
Illinois
Weeks to X Cases
	100K	200K	500K	1M
20A	14	33	--	--
20B	32	--	--	--
20C	30	--	--	--
20G	18	20	--	--
20I (Alpha)	15	--	--	--
20J (Gamma)	--	--	--	--
21A/I/J (Delta)	16	20	32	--
21K/L/M (Omicron)	3	4	6	10
Cumulative Cases by Clade (estimated, in thousands)
Weeks since Clade Arrival
United States
Weeks to X Cases
	1M	2M	5M	10M	20M
20A	8	17	34	--	--
20B	32	41	--	--	--
20C	17	33	--	--	--
20G	24	28	33	--	--
20I (Alpha)	20	24	--	--	--
21C (Epsilon)	21	25	--	--	--
21A/I/J (Delta)	16	18	21	26	--
21K/L/M (Omicron)	3	4	4	5	7
Cumulative Cases by Clade (estimated, in millions)
Weeks since Clade Arrival

Figure S2: Comparison of Cook County sequences to national and global sequences. (A) Phylogenetic reconstruction of Cook County sequences overlaid with temporally sampled national and global sequences **(B)** Frequency of clades for representative cities in the USA. **(C)** Estimated cumulative cases for each clade for Illinois and the United States. Estimated cases calculated by multiplying frequency of clades by number of cases per week. Related to Figure 2.

A

Outcome ~ Gender+ Race + Age + Comorbidities + BMI + Vaccine Doses

Variable	Coefficient	Standard Error	z	p-value	95% Confidence Interval
Intercept	-3.9641	0.182	-21.803	**0.000**	[-4.32, -3.608]
Gender- Male	0.1091	0.063	1.72	0.085	[-0.015, 0.233]
Race - Black or African American	0.5834	0.078	7.474	**0.000**	[0.43, 0.736]
Race - Other	0.8746	0.093	9.39	**0.000**	[0.692, 1.057]
Race - Declined	0.0471	0.146	0.323	0.746	[-0.238, 0.333]
Race - Asian	0.2647	0.166	1.595	0.111	[-0.061, 0.59]
Age	0.0268	0.002	12.518	**0.000**	[0.023, 0.031]
Comorbidities (sum)	0.4206	0.02	21.003	**0.000**	[0.381, 0.46]
Body Mass Index	0.0116	0.004	2.64	**0.008**	[0.003, 0.02]
Vaccine Doses before Infection	-0.6071	0.027	-22.532	**0.000**	[-0.66, -0.554]

Note: The most frequent categorical variables for gender (female) and race (white) were set as the reference.

B

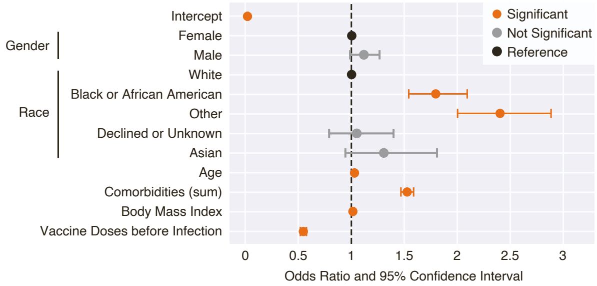

C

Outcome ~ Clade + Gender + Race + Age + Comorbidities + BMI + Vaccine Doses

Variable	Coefficient	Standard Error	z	p-value	95% Confidence Interval
Intercept	-4.0229	0.522	-7.701	**0.000**	[-5.047, -2.999]
Clade - 20B	-0.5957	0.418	-1.426	0.154	[-1.414, 0.223]
Clade - 20C	0.3195	0.375	0.853	0.394	[-0.415, 1.054]
Clade - 20G	-1.7352	0.338	-5.127	**0.000**	[-2.399, -1.072]
Clade - 20I (Alpha)	-0.0295	0.372	-0.079	0.937	[-0.759, 0.7]
Clade - 21J/I/A (Delta)	-1.2654	0.314	-4.03	**0.000**	[-1.881, -0.65]
Clade - 21M/K/L (Omicron)	-0.7895	0.352	-2.245	**0.025**	[-1.479, -0.1]
Clade - Other	-0.4884	0.365	-1.337	0.181	[-1.204, 0.228]
Gender - Male	0.3538	0.158	2.235	**0.025**	[0.044, 0.664]
Race - Black or African American	0.7572	0.206	3.672	**0.000**	[0.353, 1.161]
Race - Other	1.3089	0.231	5.666	**0.000**	[0.856, 1.762]
Race - Declined	0.5504	0.32	1.719	0.086	[-0.077, 1.178]
Race - Asian	0.5615	0.413	1.359	0.174	[-0.248, 1.371]
Age	0.0444	0.005	8.155	**0.000**	[0.034, 0.055]
Comorbidities (sum)	0.3215	0.051	6.34	**0.000**	[0.222, 0.421]
Body Mass Index	0.0183	0.011	1.743	0.081	[-0.002, 0.039]
Vaccine Doses before Infection	-0.6115	0.098	-6.23	**0.000**	[-0.804, -0.419]

Note: The most frequent categorical variables for gender (female) and race (white) were set as the reference.
Clade 20A served as the reference for the categorical variable 'clade'.

Figure S3: Elucidating the effect of clade in modeling hospitalization. (A) Parameters for the multivariable logistic regression to model hospitalized (defined as hospital admission, ICU admission and death) compared to non-hospitalized, excluding clade. **(B)** Odds ratio plot and 95% confidence interval (CI) as calculated by a multivariable logistic regression modeling hospitalized (defined as hospital admission, ICU admission and death) compared to non-hospitalized. Significant features (p-value < 0.05) are highlighted in orange. **(C)** Parameters for the multivariable logistic regression to model hospitalized (defined as hospital admission, ICU admission and death) compared to non-hospitalized. including clade. Related to Figure 3.

A

Outcome ~ Gender+ Race + Age + Comorbidities + BMI + Vaccine Doses

Variable	Coefficient	Standard Error	z	p-value	95% Confidence Interval
Intercept	-2.4084	0.345	-6.981	**0.000**	[-3.085, -1.732]
Gender - Male	0.6107	0.112	54.77	**0.000**	[0.392, 0.829]
Race - Black or African American	0.2415	0.131	1.842	0.066	[-0.016, 0.499]
Race - Declined or Unknown	0.5815	0.271	2.146	**0.032**	[0.051, 1.113]
Race - Other	0.6385	0.15	4.252	**0.000**	[0.344,0.933]
Age	0.0169	0.004	4.709	**0.000**	[0.01, 0.024]
Comorbidities (sum)	0.0992	0.03	3.254	**0.001**	[0.039, 0.159]
Body Mass Index	0.0195	0.007	2.698	**0.007**	[0.005, 0.034]
Vaccine Doses before Infection	-0.3553	0.052	-6.876	**0.000**	[-0.457, -0.254]

Note: The most frequent categorical variables for gender (female) and race (white) were set as the reference.

B

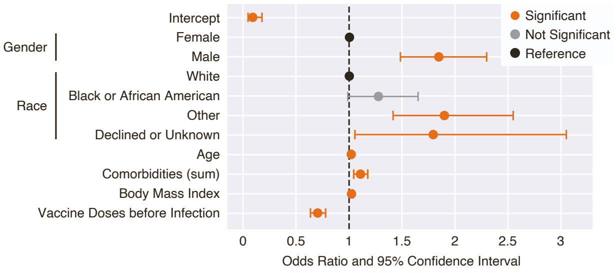

C

Outcome ~ Clade + Gender + Race + Age + Comorbidities + BMI + Vaccine Doses

Variable	Coefficient	Standard Error	z	p-value	95% Confidence Interval
Intercept	-1.3847	0.873	-1.586	0.113	[-3.096, 0.327]
Clade - 20B	0.1477	0.674	0.219	0.826	[-1.172, 1.468]
Clade - 20C	0.301	0.533	0.564	0.573	[-0.745, 1.347]
Clade - 20G	-0.6677	0.542	-1.232	0.218	[-1.73, 0.394]
Clade - 20I (Alpha)	-0.994	0.547	-1.816	0.069	[-2.067, 0.079]
Clade - 21J/I/A (Delta)	-0.8076	0.484	-1.668	0.095	[-1.757, 0.141]
Clade - 21M/K/L (Omicron)	-0.6697	0.575	-1.166	0.244	[-1.796, 0.456]
Clade - Other	-0.7309	0.533	-1.372	0.17	[-1.775, 0.313]
Gender - Male	0.4416	0.261	1.691	0.091	[-0.07, 0.953]
Race - Black or African American	0.5954	0.329	1.809	0.071	[-0.05, 1.241]
Race - Declined or Unknown	0.2532	0.528	0.48	0.631	[-0.781, 1.287]
Race - Other	0.5857	0.344	1.703	0.089	[-0.088, 1.26]
Age	0.0165	0.008	1.983	**0.047**	[0, 0.033]
Comorbidities (sum)	0.0974	0.076	1.283	0.199	[-0.051, 0.246]
Body Mass Index	0.0262	0.017	1.572	0.116	[-0.006, 0.059]
Vaccine Doses before Infection	-0.3925	0.175	-2.237	**0.025**	[-0.736, -0.049]

Note: The most frequent categorical variables for gender (female) and race (white) were set as the reference.
Clade 20A served as the reference for the categorical variable 'clade'.

Figure S4: Elucidating the effect of clade in modeling ICU admission. (A) Parameters for the multivariable logistic regression to model ICU admission excluding clade (n=1,525 inpatients). **(B)** Odds ratio plot and 95% confidence interval (CI) as calculated by a multivariable logistic regression modeling ICU admission. Significant features (p-value <0.05) are highlighted in orange. **(C)** Parameters for the multivariable logistic regression to model ICU admission including clade data (n=315 inpatients). Related to Figure 5.

A

Outcome ~ Clade + Gender+ Race + Age + Comorbidities + BMI + Vaccine Doses + Cases + Tests + Sequence/Case Ratio

Variable	Coefficient	Standard Error	z	p-value	95% Confidence Interval
Intercept	-3.2322	0.553	-5.846	**0.000**	[-4.316, -2.149]
Clade - 20B	-0.4093	0.43	-0.951	0.341	[-1.252, 0.434]
Clade - 20C	0.0571	0.392	0.146	0.884	[-0.711, 0.826]
Clade - 20G	-1.4683	0.35	-4.196	**0.000**	[-2.154, -0.782]
Clade - 20I (Alpha)	0.2083	0.383	0.544	0.586	[-0.542, 0.958]
Clade - 21A/I/J (Delta)	-0.5492	0.347	-1.583	0.113	[-1.229, 0.131]
Clade - 21K/L/M (Omicron)	0.329	0.423	0.779	0.436	[-0.499, 1.157]
Clade - Other	-0.3272	0.383	-0.854	0.393	[-1.078, 0.424]
Gender- Male	0.333	0.161	2.067	**0.039**	[0.017, 0.649]
Race - Black or African American	0.7768	0.212	3.659	**0.000**	[0.361, 1.193]
Race - Other	1.2982	0.235	5.523	**0.000**	[0.837, 1.759]
Race - Declined or Unknown	0.586	0.321	1.828	0.068	[-0.042, 1.214]
Race - Asian	0.5894	0.422	1.396	0.163	[-0.238, 1.417]
Age	0.0442	0.006	7.984	**0.000**	[0.033, 0.055]
Comorbidities (sum)	0.3377	0.052	6.495	**0.000**	[0.236, 0.44]
Body Mass Index	0.0149	0.011	1.383	0.167	[-0.006, 0.036]
Vaccine Doses before Infection	-0.6415	0.101	-6.377	**0.000**	[-0.839, -0.444]
Cases	-0.00009	0.00004	2.018	**0.044**	[0.000003, 0.0002]
Tests Administered	-0.00003	0.000006	-5.186	**0.000**	[-0.00005, -0.00002]
Sequence to Case Ratio	-0.0675	0.022	-3.085	**0.002**	[-0.11, -0.025]

Note: The most frequent categorical variables for gender (female) and race (white) were set as the reference.
Clade 20A served as the reference for the categorical variable 'clade'.

B

Outcome ~ Clade + Gender + Race + Age + Comorbidities + BMI + Cases + Tests + Sequence/Case Ratio

Variable	Coefficient	Standard Error	z	p-value	95% Confidence Interval
Intercept	-3.4992	1.743	-2.008	**0.045**	[-6.915, -0.083]
Clade - 20B	-0.1953	0.651	-0.3	0.764	[-1.471, 1.081]
Clade - 20C	-0.6145	0.702	-0.875	0.382	[-1.991, 0.762]
Clade - 20G	-0.8454	0.501	-1.687	0.092	[-1.828, 0.137]
Clade - Other	-0.0829	0.996	-0.083	0.934	[-2.035, 1.869]
Gender - Male	0.4177	0.39	1.07	0.285	[-0.347, 1.183]
Race - Black or African American	1.1326	0.51	2.222	**0.026**	[0.134, 2.131]
Race - Other	1.2086	0.553	2.186	**0.029**	[0.125, 2.292]
Race - Declined or Unknown	1.0574	0.755	1.4	0.162	[-0.423, 2.538]
Age	0.072	0.015	4.69	**0.000**	[0.042, 0.102]
Comorbidities (sum)	0.1944	0.129	1.504	0.133	[-0.059, 0.448]
Body Mass Index	0.0447	0.027	1.646	0.1	[-0.009, 0.098]
Cases	-0.0001	0.000	-0.346	0.729	[-0.0007, 0.0005]
Tests Administered	-0.00008	0.00004	-2.037	**0.042**	[-0.0002, -0.000003]
Sequence to Case Ratio	-0.4535	0.101	-4.47	**0.000**	[-0.652, -0.255]

Note: The most frequent categorical variables for gender (female) and race (white) were set as the reference.
Clade 20A served as the reference for the categorical variable 'clade'.

Figure S5: Model parameters for multivariable logistic regression modeling hospitalization while controlling for epidemiological features. (A) Parameters for the multivariable logistic regression to model hospitalized (defined as hospital admission, ICU admission and death) compared to non-hospitalized including epidemiological parameters (n=1,597). **(B)** Parameters for the multivariable logistic regression to model hospitalized (defined as hospital admission, ICU admission and death) compared to non-hospitalized including epidemiological parameters limiting to August 1st 2020 and February 1st 2021 (n=285). Related to Figure 6.

Characteristic	**Overall**, N = 14,252[a, b]	**Sequences** N = 2,114 [a, b]	**Wave 1**, N = 443[a, b]	**Wave 2**, N = 3,064[a, b]	**Wave 3**, N = 554[a, b]	**Wave 4**, N = 1,281[a, b]	**Wave 5**, N = 8,910[a, b]
Age	42(31, 57)	43 (30,58)	56(37, 67)	46(32, 60)	42(29, 55)	40(30, 57)	41(31, 56)
Body Mass Index	27(23, 31)	27 (24, 31)	29(25, 35)	27(24, 32)	27(24, 32)	26(23, 31)	27(23, 31)
Female	8,208(57.6%)	1148 (54.3%)	199(44.9%)	1,636(53.4%)	310(56.0%)	673(52.5%)	5,390(60.5%)
Male	6,044(42.4%)	966 (45.7%)	244(55.1%)	1,428(46.6%)	244(44.0%)	608(47.5%)	3,520(39.5%)
White	8,129(57.0%)	1260 (59.6%)	147(33.2%)	1,761(57.5%)	297(53.6%)	797(62.2%)	5,127(57.5%)
Black or African American	2,284(16.0%)	310 (14.7%)	164(37.0%)	443(14.5%)	105(19.0%)	176(13.7%)	1,396(15.7%)
Race Other	1,625(11.4%)	241 (11.4%)	78(17.6%)	420(13.7%)	63(11.4%)	118(9.2%)	946(10.6%)
Race Declined	1,175(8.2%)	156 (7.4%)	25(5.6%)	269(8.8%)	48(8.7%)	105(8.2%)	728(8.2%)
Asian	821(5.8%)	124 (5.9%)	19(4.3%)	147(4.8%)	23(4.2%)	71(5.5%)	561(6.3%)
Race Unknown	90(0.6%)	7 (0.3%)	8(1.8%)	5(0.2%)	16(2.9%)	11(0.9%)	50(0.6%)
American Indian or Alaska Native	55(0.4%)	6 (0.3%)	0(0.0%)	11(0.4%)	1(0.2%)	3(0.2%)	40(0.4%)
Race Unable to Answer	54(0.4%)	7 (0.3%)	0(0.0%)	1(0.0%)	0(0.0%)	0(0.0%)	53(0.6%)
Native Hawaiian or Other Pacific Islander	19(0.1%)	3 (0.1%)	2(0.5%)	7(0.2%)	1(0.2%)	0(0.0%)	9(0.1%)
Not Hispanic or Latino	10,939(76.8%)	1632 (77.2%)	294(66.4%)	2,271(74.1%)	401(72.4%)	1,018(79.5%)	6,955(78.1%)
Hispanic or Latino	1,829(12.8%)	288 (13.6%)	116(26.2%)	478(15.6%)	80(14.4%)	134(10.5%)	1,021(11.5%)
Ethnicity Declined	1,194(8.4%)	161 (7.6%)	24(5.4%)	277(9.0%)	55(9.9%)	104(8.1%)	734(8.2%)
Ethnicity Unknown	281(2.0%)	33 (1.6%)	1(0.2%)	37(1.2%)	18(3.2%)	25(2.0%)	200(2.2%)
Hypertension	3,564(25.0%)	532 (25.2%)	214(48.3%)	831(27.1%)	125(22.6%)	310(24.2%)	2,084(23.4%)
Cancer	3,347(23.5%)	480 (22.7%)	99(22.3%)	700(22.8%)	115(20.8%)	299(23.3%)	2,134(24.0%)
Cardiovascular Disease	2,755(19.3%)	424 (20.1%)	166(37.5%)	638(20.8%)	105(19.0%)	234(18.3%)	1,612(18.1%)
Asthma	1,583(11.1%)	239 (11.3%)	49(11.1%)	313(10.2%)	56(10.1%)	145(11.3%)	1,020(11.4%)
Diabetes Mellitus	1,431(10.0%)	232 (11%)	130(29.3%)	370(12.1%)	57(10.3%)	100(7.8%)	774(8.7%)
Renal Disease	1,370(9.6%)	245 (11.6%)	151(34.1%)	332(10.8%)	61(11.0%)	102(8.0%)	724(8.1%)
Immune Disorder	860(6.0%)	117 (5.5%)	31(7.0%)	171(5.6%)	25(4.5%)	83(6.5%)	550(6.2%)
Human Immunodeficiency Virus	842(5.9%)	108 (5.1%)	18(4.1%)	149(4.9%)	34(6.1%)	66(5.2%)	575(6.5%)

Chronic Liver Disease	354(2.5%)	44 (2.1%)	13(2.9%)	72(2.3%)	13(2.3%)	33(2.6%)	223(2.5%)
Chronic Obstructive Pulmonary Disease	328(2.3%)	53 (2.5%)	32(7.2%)	87(2.8%)	9(1.6%)	26(2.0%)	174(2.0%)
Solid Organ Transplant	219(1.5%)	41 (1.9%)	14(3.2%)	58(1.9%)	13(2.3%)	15(1.2%)	119(1.3%)
Unvaccinated	6,257(43.9%)	1034 (48.9%)	443(100.0%)	3,053(99.6%)	451(81.4%)	445(34.7%)	1,865(20.9%)
Partially Vaccinated	439(3.1%)	74 (3.5%)	0(0.0%)	10(0.3%)	32(5.8%)	53(4.1%)	344(3.9%)
Fully Vaccinated	4,432(31.1%)	681 (32.2%)	0(0.0%)	1(0.0%)	71(12.8%)	780(60.9%)	3,580(40.2%)
Boosted	3,124(21.9%)	325 (15.4%)	0(0.0%)	0(0.0%)	0(0.0%)	3(0.2%)	3,121(35.0%)

Table S1 | Patient Demographics by Wave, Related to Figures 3, 4, 5, 6.

[a]median (IQR) for Continous; n (%) for Categorical

[b]Cutoff dates calculated via lowest 7-day moving average of cases.

Wave 1: March 17th, 2020 – September 15th, 2020; **Wave 2**: September 16th, 2020 – March 15th, 2021; **Wave 3**: March 16th, 2021 – June 30th 2021; **Wave 4**: June 31st, 2021 – October 23rd, 2021; **Wave 5**: October 24th, 2021 – March 17th, 2022.
